# Proton-Exchange Membranes with Stabilized Conductivity

**DOI:** 10.3390/membranes16070245

**Published:** 2026-07-17

**Authors:** Andrey A. Nechitailov, Anna Krasnova, Angelina G. Kastsova, Nadezhda V. Glebova

**Affiliations:** Ioffe Institute, 26 Polytechnicheskaya, Saint Petersburg 194021, Russia; aan.shuv@mail.ioffe.ru (A.A.N.); krasnova@mail.ioffe.ru (A.K.); glebova@mail.ioffe.ru (N.V.G.)

**Keywords:** Nafion, Aquivion, proton-exchange materials, cluster-network model, moisture independence, microstructure, proton conductivity, graphene oxide, nanocomposite membrane, water retention capacity, sulfonic acid group

## Abstract

Proton-exchange membranes are crucial for water electrolyzers and fuel cells, but their performance declines under low humidity due to dehydration. Existing reviews mainly list experimental results without analyzing the mechanisms of proton conductivity stabilization. This review systematically summarizes approaches to enhance moisture-independent proton conductivity and evaluates their prospects. Key factors governing conductivity include microstructure, sulfonic group concentration, and hydration level. Stability under dry conditions depends on water retention and thermal resistance. Main strategies involve hybrid composite membranes, ionomer structure control via pre-treatments, and novel proton-conducting polymers. Promising directions include oriented channel structures, MOFs, and graphene-based materials. The stabilization mechanism relies on retaining water through hydrophilic additives that form stable hydrates, enabling proton transport even under harsh conditions (up to 120 °C and 50% RH, per US DOE targets). Among Nafion alternatives, sulfonated aromatic polymers and phosphoric-acid-doped polybenzimidazole demonstrate good performance at elevated temperatures (100–200 °C), though durability remains a challenge for the latter. Despite ongoing research, Nafion-based composites still offer one of the best overall balances of conductivity, stability, and processability. A significant research gap persists: long-term membrane performance is poorly studied, and many additives degrade over time or block proton transport sites due to ion exchange with metal cations.

## 1. Introduction

The development of technological and device solutions in the field of distributed energy is critical for the transition to clean and resource-efficient energy. The development of electrochemical energy devices, such as water electrolyzers, fuel cells, and supercapacitors, is currently of great importance. At present, proton-exchange membrane electrochemical energy conversion systems are the most widely used due to their high efficiency and operational simplicity. The operation of such devices is highly dependent on the water balance. Under low humidity conditions, membrane dehydration occurs, leading to a sharp increase in proton transport resistance and a decrease in device performance. Therefore, increasing the moisture independence of proton-exchange materials is one of the priority tasks in the field of electrochemical, particularly hydrogen, energy.

Existing reviews focus primarily on listing and describing various methods for increasing water independence and the practical results of their implementation. Despite the great value of these studies as factual material, there is a lack of analysis of the cause-and-effect relationships of each effect, an analysis of the mechanisms of proton transport stabilization using various approaches, and a summary of results. Each existing review focuses on a single aspect of the issue. Each review addresses only a particular aspect of the issue, and to date, no comprehensive analysis exists that considers the problem from multiple perspectives. Therefore, our analytical review aims to address this gap.

In this work, the approaches and technological methods that are used to increase and stabilize proton conductivity of membranes are systematized and summarized; the mechanisms and stabilization efficiency for various implementation options are analyzed. The prospects of the different methods employed are assessed.

Review articles devoted to the development of humidity-independent membranes and the investigation of various approaches used for this purpose periodically appear in the world scientific literature. At present, studies devoted to Nafion-like membranes focus on overcoming their main drawback: a sharp decrease in proton conductivity at low humidity levels and high temperatures. The goal is to create materials capable of operating effectively under so-called “humidity-independent” or “dehydrated” conditions, which simplifies humidity control systems in fuel cells and electrolyzers, for example, as reported in [1,2,3,4].

The key research trends highlighted in reviews [1,2,3,4] include:

- Nanocomposite membranes. The majority of current research focuses on the incorporation of different fillers, including inorganic nanoparticles, graphene oxide (GO), and proton-conducting materials, into the Nafion matrix. These fillers help retain bound water or create additional channels for proton transport even under low-humidity conditions, thus increasing device performance.
- Surface modification. Methods of surface engineering (creation of micro- and nanostructured patterns) of Nafion membranes are being studied to optimize the three-phase boundary, improve water management, and increase proton transport.
- New alternative materials. Active research continues on alternative materials, such as sulfonated aromatic polymers (for example, sulfonated poly(ether ether ketone) (SPEEK)), which have the potential to replace Nafion, as they offer operation at high temperatures without intensive hydration and have lower costs.
- Applications and efficiency. Current reviews analyze the use of modified membranes in high-temperature proton-exchange membrane fuel cells (HT-PEMFCs) and water electrolyzers, evaluating their performance, durability, and economic feasibility compared to standard Nafion.

The main conclusion that can be drawn from recent review studies is as follows: although pristine Nafion membranes exhibit a notable decline in performance at low humidity levels, the development of Nafion-based nanocomposite membranes demonstrates promising results, providing more stable and higher efficiency under harsh operating conditions (up to 120 °C and 50% RH, per US DOE targets).

It is important to note that in these reviews, attention is mostly focused on listing and describing various techniques for increasing humidity independence and practical results from their implementation. Despite the great value of these (and other) similar works as factual material, there is a lack of analysis of the cause-and-effect relationships of particular effects, analysis of the mechanisms stabilizing proton transport when using different techniques, and generalization of results. Each of the existing reviews is devoted to considering some single aspect of the issue, and to date, there is no generalized analysis addressing the problem from multiple perspectives. Consequently, the purpose of our analytical review is to address this gap.

## 2. Review Methodology

In shaping the concept of this review, the authors aimed to systematize a vast body of literature and present it in a form accessible to both experts and researchers entering the field.

The selection and analysis of the literature were governed by the following criteria:

1. Core Fundamentals: Consideration of the fundamental nature of proton exchange membranes (PEMs), including their composition, structure, properties, ion transport mechanisms, and general challenges associated with proton conductivity.
2. Problem-Oriented Approach: Analysis of the key factors driving material degradation and modern stabilization strategies. Specific emphasis was placed on the degradation of critical parameters such as ionic resistance (conductivity) and membrane durability under harsh operating conditions (up to 120 °C and 50% RH, per US DOE targets).
3. Mechanistic Depth: Priority was given to studies that moved beyond merely reporting empirical data to explore the underlying relationships between composition, structure, processing technology, and stabilization mechanisms.
4. Conceptual Selection: Articles were chosen for citation if they served as representative and illustrative benchmarks for specific scientific and technological paradigms.

This work does not intend to provide an exhaustive, cataloged compendium of every existing stabilization technique in global practice. Instead, it aims to synthesize the accumulated data to highlight key conceptual trends and the most effective contemporary strategies in the field.

The primary literature corpus covers the last 5–7 years, a timeframe selected due to the rapid accumulation of qualitatively new global insights that have not yet been adequately synthesized. Older, pioneering publications were included selectively, strictly based on their foundational significance to the subject matter.

The primary thematic accent of this review is placed on the utilization of interfacial interactions (e.g., the formation of surface compounds, structural interpenetration, and strong adsorption) to modulate material properties. This direction is identified as a highly versatile and promising tool for the effective tailored management of PEM characteristics and strength.

## 3. Proton-Exchange Membranes: A Brief Overview

Owing to its favorable combination of physicochemical properties, Nafion and Nafion-like ionomers [5,6,7,8] are currently recognized as the benchmark material for ion-exchange membranes in electrochemical energy conversion systems. What are these properties? Among the key features of these polymers, one can include (in order of importance) strong acidic properties, relatively high proton conductivity, low gas permeability, chemical stability, thermal stability, and adequate mechanical strength.

These materials are perfluorinated polyethers containing sulfonic (–SO_3_H) groups of atoms. These are Bronsted strong acids with a large polymeric acid residue. The presence of a hydrophobic fluorocarbon backbone and hydrophilic sulfonic groups imparts these compounds surface-active properties and the ability to form micelles. The fluorocarbon backbone (due to the very high electronegativity of fluorine) provides high chemical inertness, thermal stability at relatively high (~250–300 °C) temperatures, and mechanical strength.

**Figure 1:**
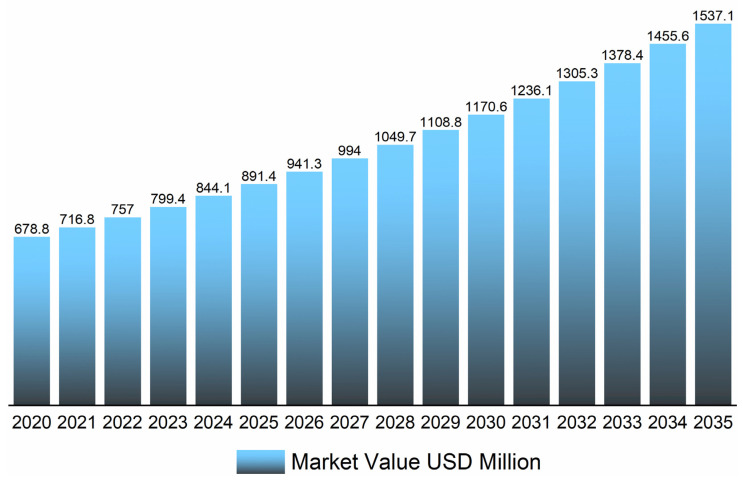
Forecasted Size of the Nafion Market for 2020–2035. Data retrieved from [9].

Due to the unique properties of Nafion-like compounds, since the discovery of Nafion in the 1960s, these materials have remained the foundation of proton-exchange membranes, and moreover, their production continues to grow (Figure 1).

Alongside the advantageous properties that make Nafion-like materials highly demanded and promising, their limitations requiring improvement are also well known. Such properties include the strong dependence of proton conductivity on humidity and temperature, which prevents efficient membrane operation at temperatures above ~100 °C, despite their thermal stability extending to higher temperatures at which catalytic processes proceed more efficiently (reaction rates increase and catalyst resistance to catalytic poisons enhances).

An analysis of the scientific literature indicates that since the discovery of Nafion, despite numerous ongoing attempts to replace it with other materials possessing improved properties, it continues to maintain a leading position due to its unique combination of characteristics [10,11,12]. At the same time, the most substantial practical results have been achieved through approaches involving the modification of Nafion, either by introducing various additives into its structure or by altering its structure through different processing methods.

A clear understanding of the nature and mechanisms of ionic (proton) conductivity is crucial for its purposeful improvement and stabilization under harsh conditions (up to 120 °C and 50% RH, per US DOE targets).

## 4. Structure and Conductivity of Nafion-like Polymers

Nafion and Nafion-like polymeric materials, such as, for example, M(L)F4-SK (Plastpolymer, St. Petersburg, Russia), Aquivion (Solvay, Spinetta Marengo, Alessandria, Italy), Flemion (Asahi Glass Co., Tokyo, Japan), and others, show the ability for cation-exchange capability, which enables their use as proton-conducting components of electrodes and proton-exchange membranes in electrochemical devices and mixed-conductivity systems. Such systems include, for example, hydrogen and methanol fuel cells, water electrolyzers, proton supercapacitors, and Red-Ox batteries. A representative Chinese commercial alternative, the Dongyue series, has recently been evaluated by Meng et al. [13], who reported a proton conductivity of 0.371 S·cm^−1^ (80 °C, 100% RH) for a 25 μm membrane, which is 139% higher than that of Nafion 211 (0.155 S·cm^−1^ under the same conditions), and a peak power density of 852 mW·cm^−2^ in a PEMFC single-cell mode, which is 34% higher than the 636 mW·cm^−2^ achieved with Nafion 211.

The membranes prevent the transfer of electrons and reactants (hydrogen, methanol, oxygen, etc.) between the anode and cathode compartments while enabling the transport of hydrated protons. Their functional purpose is to enable proton transport. An important requirement for the membrane is its high and stable ability to transport protons over a wide range of conditions. Due to their organic nature, these membranes exist within a rather narrow temperature interval, the upper limit of which is approximately 250–300 °C. However, the membrane loses its performance at even lower temperatures, ~100–120 °C, due to dehydration, since proton conductivity occurs only in the presence of a sufficient amount of water in the membrane structure. Proton conductivity is highly sensitive to membrane humidity and drops sharply as humidity decreases. Accordingly, not only temperature but also low ambient humidity has a detrimental effect on proton conductivity. Considering the temperature ranges of the physical existence of the polymeric materials under study, it is evident that their potential operating capabilities extend by 100–150 °C toward higher temperatures. Thus, the key issue is not physical degradation of the material, but rather a decrease in conductivity under dehydration conditions. Hence, it is reasonable to develop humidity-independent proton-exchange materials.

To eliminate qualitative ambiguity and ensure an objective comparative analysis of the discussed modification methods, it is essential to establish precise quantitative criteria for the terms “humidity-independent” and “moisture-independent” proton conductivity. Following the stringent performance targets set by the U.S. Department of Energy (US DOE) for HT-PEMFCs, a membrane material is classified as technologically viable under non-humidified conditions if it can provide an absolute proton conductivity (σ) of at least 0.01 S·cm^−1^ (10 mS·cm^−1^) at an elevated temperature of 120 °C and a sub-stoichiometric relative humidity (RH) of 50% [14].

Furthermore, in the low-temperature operating range (up to 80–100 °C), the concept of “humidity independence” is quantitatively defined by a suppressed operational decay coefficient, where the overall decrease in conductivity should not exceed 1–1.5 orders of magnitude upon an aggressive drop in relative humidity from a fully hydrated state (95% RH) to harsh “dry” operating conditions (30% or 20% RH) [15]. 

Having established these quantitative benchmarks, we now turn to the fundamental origins of proton transport. The transfer of protons in these membranes is provided by acidic sulfonic groups –SO_3_H, present in the composition of the membrane material. The microstructure of the ionomer plays a decisive role in proton conductivity. Key factors governing proton conductivity include the microstructure of the ionomer, the density (concentration) of sulfonic groups (determined by the equivalent weight, EW), and the degree of hydration of the sulfonic groups (water content). Proton conductivity resilience to low humidity and high temperatures is determined by water-holding capacity and thermal stability.

Historical understanding of the nanostructure of perfluorosulfonic acid (PFSA) ionomers was based on classical phenomenological models. The classic cluster-channel model by Gierke [16], based on small-angle X-ray scattering (SAXS) data, postulated the presence of spherical inverted micelles approximately 4 nm in diameter connected by narrow pores (~1 nm). Through the fundamental works of Ozerin, Rebrov [17,18], Bakeev, and Yaroslavtsev [19], these concepts were significantly advanced, providing a detailed description of macromolecular aggregation in solutions and the morphological rearrangements of PFSA matrices during oriented drawing and saponification. However, these early models, including the elongated polymer aggregate concept of Rubatat [20] and the rigid ribbon-like model of Kreuer [21], were largely idealized due to the limitations of ex situ characterization methods.

Studies employing cryo-electron tomography and high-resolution microscopy (cryo-TEM/cryo-ET) [22] have experimentally demonstrated that the actual morphology of ionomer films does not exhibit well-defined spherical clusters. Recent in situ small-angle X-ray scattering (SAXS) experiments, together with molecular dynamics simulations of hydration processes [23,24], have made it possible to quantitatively establish that upon drying (λ < 3), what occurs is not merely a decrease in pore radius but rather a local collapse and percolation-driven disruption of channel connectivity.

The introduction of nanocomposite and hybrid additives fundamentally alters membrane topology, which is now successfully visualized using local interface analysis techniques. Solid-state magic-angle spinning nuclear magnetic resonance (^1^H MAS NMR) enables quantitative assessment of the contribution of interfacial interactions at the ionomer/additive boundary. Experimental evidence proves that the incorporation of modified silicates (sSLM) results in strong interfacial anchoring [3]. Characteristic chemical shifts and changes in proton spin–lattice relaxation times indicate the formation of a dense hydrogen-bond network between the acidic groups of the additive and the hydration shell of the Nafion sulfonate groups. This interfacial constraint severely suppresses the mobility of the local proton subsystem and stabilizes the geometry of hydrophilic domains, preventing excessive shrinkage of conductive channels under dehydration conditions.

In summary, modern physical in situ characterization methods have proven that effective stabilization of proton pathways in hybrid membranes is achieved not by extensive channel expansion, but rather by local thermodynamic densification of hydrogen bonds at the interface and the retention of percolation connectivity of ionic pathways upon dehydration.

A fundamental understanding of why these modification strategies succeed or fail under low humidity conditions requires a quantitative framework. Such a framework is provided by the balance between the two microscopic proton transport mechanisms: the Grotthuss (hopping) mechanism and the vehicular (diffusive) mechanism. According to molecular dynamics (MD) simulations, the contribution of each mechanism is strictly determined by three interrelated parameters: water activity (a_w_), pore geometry, and local hydrophilicity [21,25,26].

The total proton conductivity (σ) can be expressed as the sum of two independent contributions: one from the Grotthuss (hopping) mechanism and the other from the vehicular (diffusive) mechanism.

The ratio of these contributions critically depends on the local channel architecture and the hydration parameter λ (number of water molecules per sulfonic acid group). Based on a synthesis of computational and experimental data, a clear threshold behavior of proton transport emerges at different humidity levels:

1. Extremely low hydration regime (λ < 3, percolation threshold) [22,26,27,28]:

In this region, water activity within the pores is minimal (a_w_ < 0.2). Water molecules are tightly bound in the primary hydration shell of the sulfonic acid groups (–SO_3_H). MD simulation data show that the activation energy for proton transfer is maximal here (E_a_ > 0.4–0.5 eV). Vehicular transport is impossible because free hydronium ions are absent. Transport occurs exclusively via rare Grotthuss hops between isolated sulfonic acid groups, and conductivity remains at a critically low level (10^−4^–10^−3^ S·cm^−1^), representing classical percolation threshold behavior.

2. Intermediate regime (3 < λ < 6, competitive transport):

As λ increases toward 6, a layer of so-called “intermediate” (bound) water forms. The activation energy decreases to 0.25–0.35 eV. Active competition between the two mechanisms begins:

Increased hydrophilicity due to the introduction of additives (e.g., TiO_2_PO_4_ nanoparticles, sSLM, sGO) locally increases water activity (a_w_) and reduces vapor pressure, forcing water molecules to remain near interfaces.

This stabilizes a continuous hydrogen-bonded network, activating the Grotthuss mechanism. Proton hopping occurs through chains of Bjerrum defects with the formation of Zundel (H_5_O_2_^+^) and Eigen (H_9_O_4_^+^) ions. The contribution of the Grotthuss mechanism in this regime reaches 60–70%, providing a sharp (exponential) increase in conductivity on the σ = f(λ) curve to levels of 10^−2^ S·cm^−1^.

3. High hydration regime (λ > 6, bulk water dominance) [21]:

At λ > 6, “free” (bulk) water appears in the pore centers (a_w_ → 1). The activation energy drops to a minimum (~0.1–0.15 eV). Channels expand and widen (to 4–5 nm for Nafion). Under these conditions, the vehicular mechanism begins to dominate—physical diffusion of protons as solvated complexes within the free volume of the pores—providing maximum conductivity (>10^−1^ S·cm^−1^).

## 5. Current Research Trends and Achievements

Numerous studies have focused on expansion of operating conditions of proton exchange membranes (increasing the operating temperature and reducing humidity) by enhancing their water-independence and thermal stability [14,27,28,29,30,31,32,33,34,35]. Three main approaches to enhancing the water-independence of membranes:

- Formation of hybrid composite membranes;
- Control of the ionomer structure through various pretreatment methods (thermal treatment, use of different solvents during membrane casting, and others);
- The creation of new proton-conducting polymers.

A separate line of work, critical for assessing the practical potential of these directions, concerns the development of standardized protocols for evaluating long-term membrane durability, including accelerated stress testing (AST).

### 5.1. Key Parameters for Proton-Exchange Membrane Durability Assessment

#### 5.1.1. Hydration State

In the literature on proton-conducting membranes, the hydration state is conventionally quantified by the water-to-sulfonic acid ratio, λ (the number of water molecules per sulfonic acid group, –SO_3_H). In [36], infrared spectroscopy clearly distinguishes water states: up to λ = 3 (bound water), λ = 6 (intermediate water), and above (free water). For PFSA membranes such as Nafion and Aquivion, lambda values of 3 and 6 represent critical thresholds that correspond to fundamental transitions in the proton transport mechanisms:

1. Critical Level λ = 3 (The Conductivity Threshold)

This stage corresponds to primary hydration, where water molecules are strongly bound to the –SO_3_H groups. At λ < 3, the membrane exhibits negligible ionic conductivity because the water molecules remain isolated from one another. Upon reaching lambda = 3, percolation of the hydration shells occurs, establishing continuous pathways for proton diffusion [37].

2. Critical Level λ = 6 (Transition to Bulk-like Water)

This level is considered the threshold at which ‘free’ (bulk) water begins to appear in the membrane pores. Below λ = 6, water is considered ‘bound water.’ Beyond this value, the channels widen sufficiently to activate the Grotthuss mechanism (proton hopping), which sharply increases proton mobility and, consequently, conductivity. In [38] it is shown that the introduction of even a small amount of water (i.e., λ~2) leads to complete dissociation of the sulfonic acid groups in the acid and deuterated forms of Nafion. Overall, the vibrational spectra become more similar as the water content increases. This is interpreted as the formation of primary cation solvation shells (i.e., [H_3_O^+^⋯(H_2_O)_n_], [D_3_O^+^⋯(D_2_O)_n_], and [Na(H_2_O)_n_]^+^) and their coordination with the terminal groups of the side chains.

#### 5.1.2. Accelerated Stress Testing (AST)

In international practice (according to US DOE and M2FCT standards, membrane stability is evaluated using accelerated stress tests [39,40,41]. These tests are conducted along two directions:

- Chemical stability (open-circuit voltage (OCV) hold/Fenton test)
  Maintaining the membrane at open-circuit voltage at elevated temperature (90 °C) and low humidity (30% RH), where damaging radicals •OH and •OOH are generated. Degradation indicators: fluoride emission rate (FER) and voltage drop. membranes.
- Mechanical stability (Relative Humidity Cycling)
  Humidity cycling (e.g., 0% RH for 2 min → 100% RH for 2 min) to simulate start/stop cycles. This causes anisotropic swelling, shrinkage, and the formation of microcracks. Degradation indicator: the number of cycles until the appearance of through-hole perforation (hydrogen gas crossover) [42].

To move from basic electrochemical characteristics to the analysis of membrane durability, it is necessary to examine the materials under study through the lens of standardized AST protocols, such as those of the US DOE. The long-term stability of both pristine PFSA matrices and modified composite membranes is fundamentally determined by two competing degradation mechanisms: chemical degradation and mechanical fatigue.

Chemical stability is typically assessed under OCV hold conditions at elevated temperatures (e.g., 90 °C) and low relative humidity (e.g., 30% RH). Such conditions sharply accelerate the electrochemical generation of highly reactive oxygen species—in particular, hydroxyl (•OH) and hydroperoxyl (•OOH) radicals—which attack the polymer backbone and terminal carboxyl groups. In unmodified Nafion or Aquivion systems, this aggressive chemical attack triggers radical degradation (“unzipping” of polymer chains), leading to local membrane thinning, catastrophic loss of functional sulfonic groups, and an exponential increase in FER.

Mechanical durability is investigated using rapid relative humidity cycling (RH cycling) protocols, for example, by alternating between 0% and 100% RH every 2 min under nitrogen purge without electrical load. Dynamic hygrothermal cycling subjects the constrained membrane to strong anisotropic swelling and shrinkage stresses. This continuous mechanical deformation leads to viscoplastic fatigue, structural relaxation, and local microcracking at interfacial boundaries. The end-of-life criterion is typically defined by a sharp increase in hydrogen crossover or electrical short circuit, signaling the formation of through-holes (perforations).

Current literature data emphasize that although strategies such as physical mixing with additives or solvent tuning successfully increase initial proton conductivity, they often exhibit opposite (negative) trends during prolonged AST. For example, composite membranes modified with unanchored hydrophilic nanoparticles demonstrate a sharp drop in stability during extended humidity cycling due to intensive additive leaching and the formation of interfacial voids. Thus, to become mature technological solutions, newly developed membranes must achieve a delicate balance between chemical resistance (suppression of FER) and mechanical strength (ability to withstand more than 20,000 humidity cycles), rather than being limited to optimizing solely initial proton conductivity.

### 5.2. Hybrid Membranes

The most common approach consists of two closely related paths: the formation of membranes filled with inorganic compounds and the introduction of organic materials into the membrane. Analysis of the literature data indicates that the most frequently used method is the creation of composite (filled) membranes by introducing hydrophilic, water-retaining materials with nanometer-sized particles, such as silica, titanium dioxide, GO, detonation nanodiamonds, and others.

The incorporation of a hydrophilic nanostructured additive into the membrane serves two purposes: increasing the ability to retain water and modifying the structure (increasing the diameter of proton-conducting channels, formation of regular structures, decreasing the tortuosity of channels).

The mechanism of enhancing water-retention ability in this case consists of the formation of hydrogen bonds and other strong interactions between the additive and the membrane matrix, as a result of which the equilibrium vapor pressure of water decreases and, consequently, water-independence increases.

A review published eleven years ago [19] describes the principal approaches to the modification of ion-exchange materials, primarily focused on the development of hybrid membranes containing inorganic nanoparticles. It was noted that the main reason for the enhanced conductivity and selectivity of hybrid membranes is the modification of pore and channel structure as well as the distribution of charge carrier concentration within them.

Since that time, the main approaches to membrane modification have not changed markedly. Safronova et al. [43] noted that one of the most prominent practical applications of PFSA membranes today is their use in fuel cell development. However, certain drawbacks of PFSA membranes, such as low conductivity at low humidity and elevated temperatures, limit their application. Property optimization strategies are based on modifying commercial membranes and PFSA polymers by introducing various additives or preliminary treatment. Based on commercially available samples, Safronova et al. [43] summarized approaches to membrane modification that enable the development of materials with a range of functional properties, differing in their ion transport characteristics (primarily proton conductivity) and selectivity.

These approaches include the use of various processing methods, as well as the creation of hybrid materials containing nanoparticles of doping additives. It has been shown that modification of the intrapore space of the membrane is a way to influence the key functional properties of membranes.

Thus, the use of fillers for the fabrication of composite (filled) membranes has proven to be a method for increasing resistance to water loss and thermal stability [3,14,27,28,29,30,31,32,44,45].

Sun et al. [14] analyzed the results of using various additives in commercially available Nafion membranes. It was highlighted that among the many studied systems, several promising materials were identified in which Nafion is combined with MOFs as well as GO, which exhibit relatively high conductivity even at 120 °C under low relative humidity (RH). The Nafion–SZM (Zr-MOF) hybrid membrane exhibits a 23% higher proton conductivity, specifically 2.96 mS·cm^−1^, compared to 2.29 mS·cm^−1^ for the unmodified Nafion membrane [46]. The Nafion–SZM membrane also demonstrates higher performance at 35% RH than Nafion, achieving about 370 mA·cm^−2^ at 0.6 V versus approximately 250 mA·cm^−2^ for Nafion. The main issue is that these membranes have not been tested in full PEMFCs, and their stability during operation has not been evaluated.

Ibrahim et al. [27] fabricated and characterized GO–Nafion composite membranes of varying thicknesses under automotive operating conditions at standard and intermediate temperatures. The composite membrane demonstrated higher mechanical strength, improved water uptake, and enhanced performance. It was shown that the use of GO–Nafion composite membranes enables up to a 20% increase in maximum power density at all operating temperatures. For example, at 80 °C, the GO–Nafion membrane with a thickness of 30 µm achieves 0.48 W·cm^−2^, whereas unmodified Nafion achieves 0.37 W·cm^−2^. In addition, the GO–Nafion membrane was able to maintain its open-circuit voltage at elevated temperature and reduced thickness, indicating improved durability and a potentially longer service life.

Rambabu et al. [28] investigated the effect of different forms of carbon nanomaterials as additives in Nafion membranes on the performance of direct methanol fuel cells (DMFCs). It was shown that carbon nanomaterials significantly influenced the development of polymer electrolytes for DMFCs. The performance of composite membrane electrolytes depends on the type of carbon nanomaterial employed, including GO (two-dimensional), carbon nanotubes (CNTs), carbon nanofibers/graphene nanofibers (one-dimensional), and fullerene. The structural orientation and composition of these carbon materials in the membrane matrix have a strong impact on membrane properties. Surface functionalization of these materials imparts hydrophilic character, finely tunes composite conductivity, and ensures better interfacial interaction with polymer matrices. For Nafion membranes, GO is the most suitable nanofiller, where reducing methanol permeability is of primary importance; the sheet-like structure of graphene is more effective than the tubular structure of CNTs in reducing fuel crossover, while CNTs are more effective in enhancing ionic conductivity.

The alignment of proton-conducting channels via CNT incorporation is a key feature of the studied composite membranes [29]. The study demonstrates that the multilayer structure in composite membranes, particularly in ultrathin membranes (1 μm), promotes the lateral alignment of sulfonated CNTs and proton pathways (parallel to the membrane interface). Furthermore, due to the formation of a hydrophilic interface between the layers, the composite membranes exhibit higher water uptake. In particular, the proton conductivity of 80-layer membranes reaches 0.33 S·cm^−1^ at a high temperature of 152 °C. Laterally aligned sulfonated CNTs result in substantially improved mechanical properties of multilayer composite membranes, leading to enhanced electrochemical stability and long-term performance stability.

Primachenko et al. [32] focused on the incorporation of detonation nanodiamond (DND) additives to enhance the performance of Aquivion-type membranes. Composite proton-conducting membranes based on perfluorinated copolymers of the Aquivion type, modified with positively charged DNDs, were prepared to enhance the performance of hydrogen fuel cells. Small-angle neutron scattering experiments revealed a fine structure in these DND-filled membranes (0–5 wt.%), where the proton-conducting channels typical of Aquivion membranes are largely preserved, while DND particles (4–5 nm in size) decorate polymer domains at the submicron scale, according to scanning electron microscopy (SEM) data. As the DND content increased (0, 0.5, and 2.6 wt.%), thermogravimetric analysis, potentiometry, and potentiodynamic and potentiostatic curves showed the stabilizing effect of DND on the operational characteristics of the membranes. Membrane–electrode assemblies (MEAs) operating in an O_2_/H_2_ system with membranes of different modified compositions demonstrated improved functional properties, such as greater operational stability, enhanced proton transport durability, and higher current density at elevated temperatures over an extended temperature range (22–120 °C) compared to pristine membranes without additives.

Sulfonated layered silica material (sSLM), when added to Nafion membranes, significantly improved ion-exchange capacity, water uptake, and dimensional stability [3]. The nanocomposite membranes demonstrated enhanced proton conductivity, particularly under harsh conditions of 120 °C and 20% RH, where unfilled Nafion largely loses conductivity: the sSLM membrane achieves 30 mS·cm^−1^, whereas Nafion achieves only 1.2 mS·cm^−1^. Single-cell tests in H_2_/O_2_ fuel cells confirmed these improvements, with the optimal sSLM–Nafion nanocomposite membrane achieving a twofold increase in power density compared to pristine Nafion at 120 °C and 20% RH (340 mW·cm^−2^ and 117 mW·cm^−2^ for Nafion, respectively).

Molybdenum dioxide (MoO_2_) nanosheets could be successfully incorporated into the Nafion membrane [44] because MoO_2_ is able to act as an effective free radical scavenger and form hydrogen bonds with Nafion chains. The Nafion–MoO_2_ composite membrane exhibits substantial improvements in both oxidative stability, with a 56.7% increase, and proton conductivity, with a 26% increase compared to pristine Nafion membranes.

Nafion nanocomposite membranes with sulfonated Clay-CNTs (sCC) additives demonstrated markedly higher proton conductivity, especially under low RH conditions, which is a critical factor for high-temperature fuel cell operation [45]. The sCC nanohybrid exhibits a conductivity of approximately 230 mS·cm^−1^ at 100% RH and 42.3 mS·cm^−1^ at 20% RH, while Nafion shows 180 mS·cm^−1^ at 100% RH and only 1.3 mS·cm^−1^ at 20% RH. Electrochemical evaluation in an H_2_/O_2_ fuel cell showed nearly a fourfold increase in peak power density (443.2 mW·cm^−2^) under harsh high-temperature, low-humidity conditions (120 °C, 20% RH) compared to reprocessed Nafion (117.3 mW·cm^−2^). 

Composite membranes with sulfonated organic frameworks incorporated into Nafion demonstrated enhanced water-independence and proton conductivity [47]. The NUS-9/Nafion composite membrane achieved a maximum power density of 1.024 W·cm^−2^, an 80% increase over pristine Nafion, and exhibited improved durability, underscoring the considerable potential of this type of membrane for PEMFC applications. The 0.5 wt% NUS-9/Nafion membrane achieves a proton conductivity of 0.207 S·cm^−1^ at 95 °C and 100% RH, whereas the unmodified Nafion membrane shows 0.11 S·cm^−1^ under the same conditions, representing an 88% increase. 

A separate, relatively recent research direction concerns the use of carbon-based A separate, relatively recent research direction concerns the use of carbon-based additives in Nafion composite proton-conducting membranes to enhance performance parameters such as high-temperature water retention, ionic resistance (conductivity), and thermal stability [14,27,28]. Commonly used additives include CNTs [24,25] and graphene-based materials [28,30,48]. 

The development of alternatives to Nafion has become one of the most critically discussed topics in the field of proton-exchange membranes in recent years [49,50,51].

One of the promising and relatively new approaches to improving ionomer stability is the use and enhancement of interfacial interactions between components. The creation of adsorptive and chemical surface bonds aims to increase corrosion resistance, structural stability, and expand the operating temperature range [52,53,54,55,56]. These studies investigated the interaction of the proton-exchange polymer Nafion with the surfaces of various carbon materials: carbon black, multi-walled CNTs, graphene, and platinum. A significant stabilizing effect of carbon materials with a developed surface (graphene) on the thermal stability of the polymer was demonstrated. The Nafion–C interfaces were studied using NMR and X-ray photoelectron spectroscopy, revealing strong interactions at the level of surface chemical bond formation.

The segregation of fillers negatively affects the spectrum of properties of the composite polymer, especially its water retention and high-temperature functionality.

Woo et al. [15] highlighted that creating a uniform structure of sepiolite filler, grafted with fluorine-containing groups, in an Aquivion-type membrane increases the homogeneity of the composite membrane prepared with Aquivion compared to composite membranes made with natural sepiolite and improves their resistance to low humidity.

The development of hybrid composite membranes incorporating diverse hydrophilic additives has proven effective in maintaining high proton conductivity under challenging conditions (up to 120 °C and 50% RH, per US DOE targets). Moreover, this approach is technologically straightforward, typically involving either the simple mixing of the ionomer solution with the additive prior to membrane casting or the impregnation of a pre-formed membrane with the additive solution.

The strategy of developing hybrid composite membranes incorporating various hydrophilic additives has demonstrated high efficiency in stabilizing proton conductivity under harsh conditions (up to 120 °C and 50% RH, per US DOE targets). Furthermore, this approach is highly advantageous due to its technological simplicity, as it often involves either straightforward blending of the ionomer solution with the additive prior to membrane casting, or the post-casting impregnation of a pre-formed membrane with an additive solution.

In this case, property stabilization is governed by the synergy of several distinct mechanisms:

- A reduction in water vapor pressure within the composite membrane due to water binding and hydrogen bond formation with the surface groups of the additive;
- Tailored rearrangement of the water channel architecture within the polymer matrix induced by the incorporation of additive nanoparticles;
- 25CFAn increase in the density of the membrane’s water channels resulting from Van der Waals interactions between the polymer chains and the embedded additive.

However, despite the high initial performance, the technological simplicity of basic blending or impregnation imposes severe constraints on the long-term durability of such systems. Under operational fuel cell conditions, continuous electro-osmotic drag and thermal cycling inevitably trigger the leaching of hydrophilic additives that lack robust chemical anchoring to the polymer matrix. This leads to a progressive degradation of proton conductivity and mechanical cracking at the interfacial boundaries. Consequently, to ensure extended durability (exceeding 500–1000 h), simple physical modification must be succeeded by strategies that induce covalent coupling or strong interfacial interactions, which are critically evaluated in the subsequent sections.

Key studies on PEM degradation highlight that hydrophilic additive leaching is caused by electro-osmotic drag, notably documented by Colicchio et al. and Kusoglu & Weber [57,58,59]. Furthermore, research by Sahu et al. demonstrates that implementing covalent interfacial bonding, rather than simple physical mixing, is essential to prevent filler migration and ensure long-term stability exceeding 1000 h. Review comprehensive membrane transport and durability studies at [60].

#### 5.2.1. Critical Analysis of Long-Term Stability of Modified Membranes

Analysis of the long-term stability of PEMs modified using various scientific and technological approaches reveals a fundamental and expected trade-off between the improvement of initial transport properties and the preservation of operational characteristics over extended periods [43]. Depending on the nature of the modifying agent and the method of its integration into the polymer matrix, degradation processes follow several scenarios.

1. Leaching problem with physical mixing

The classical approach, involving the simple introduction (doping) of hydrophilic nanoparticles or heteropolyacids into the Nafion matrix or into high-temperature PEMs, exhibits pronounced instability under dynamic operating conditions [27,43].

Degradation mechanism: The presence of functional additives that are “free” (chemically unbound to the polymer) leads to their gradual diffusion and leaching by liquid water flows generated at the cathode, as well as by electro-osmotic drag [27].

Consequences: Durability tests show that the loss of dopants leads not only to a sharp drop in proton conductivity (due to a decrease in the λ parameter) but also to the formation of internal voids at interfacial boundaries. These microvoids become centers of mechanical stress concentration, provoking accelerated membrane cracking during humidity cycling [27,43].

2. Structural lability under solvo-thermal tuning

Modification of ionomer morphology by selecting casting solvents, introducing plasticizers, or varying drying regimes makes it possible to optimize channel packing and increase the initial current [14,32,43].

Degradation mechanism: Perfluorinated macromolecules in such systems exist in a thermodynamically non-equilibrium (labile) state [32,43]. At elevated operating temperatures (especially above 80–90 °C) and under conditions of constant hydration changes, macromolecular relaxation processes are activated [14].

Consequences: The structure tends to return to its original stable state with minimal free energy. This causes gradual shrinkage of the conducting channels, local collapse of hydrophilic domains, and an irreversible decline in water retention capacity in the long term [32].

3. Thermomechanical stabilization by inorganic frameworks and clays

The introduction of layered silicates, modified clays (e.g., sulfonated layered materials, sSLM), and carbon nanotubes represents a qualitatively different level of stabilization [3,29,45].

Stabilization mechanism: As shown in works from 2025 [29,45], the use of sSLMs and clay/carbon nanotube nanocomposites provides a strong synergistic effect. Rigid inorganic nanolayers create a spatial framework that prevents excessive anisotropic swelling and shrinkage of the membrane during sharp humidity fluctuations.

Long-term effect: Covalent or strong electrostatic anchoring of sulfonic groups to the inorganic phase completely solves the leaching problem. Such systems demonstrate excellent thermomechanical stability under harsh operating conditions (up to 120 °C and 50% RH, per US DOE targets) by minimizing viscoplastic fatigue of the polymer matrix and preventing the initiation of microcracks at interfaces [29,45].

4. Crosslinking effect and carbon nanomodifiers

The use of covalent crosslinking, incorporation of graphene structures, graphene oxide, and functionalized carbon frameworks makes it possible to rigidly fix the architecture of the conducting pathways [28,30,31,44].

Stabilization mechanism: A three-dimensional entanglement network or strong interfacial adsorption interaction (especially at phase boundaries) limits the mobility of Nafion backbone segments [28,44]. Crosslinked structures and composites with carbon nanomaterials effectively suppress the mobility of polymer molecules under cyclic heating [30,31].

Long-term effect: Restricting the conformational mobility of the polymer prevents structural relaxation and “unzipping” of chains under the action of Fenton radicals (•OH, •OOH). This sharply reduces the FER and prevents membrane thinning, placing these approaches among the most promising for achieving fuel cell lifetime targets [31,44].

Thus, comparative durability analysis shows that the most viable stabilization strategies are those based not on simple physical encapsulation of additives, but on the formation of extended interfacial interactions (as in the case of sulfonated layered clays and carbon nanotubes [3,45]) or the creation of three-dimensional crosslinked polymer networks [26,39].

#### 5.2.2. Quantitative Durability Assessments of Modified Membranes [3,14,27,28,29,30,31,32,43,44,45]

Leaching intensity and conductivity loss: For composites obtained by simple physical blending of unanchored hydrophilic nanoparticles or heteropolyacids, the critical point of degradation occurs after only 100–200 h of continuous operation. During this period, due to additive leaching, proton conductivity drops by 30–50% of its initial value.

Chemical degradation (OCV hold and radical attack): During standard OCV tests (at temperatures of 90–120 °C), the fluoride emission rate (FER) for modified systems without interfacial anchoring sharply increases, reaching values on the order of 10^−5^–10^−4^ mg/cm^2^·h. This indicates accelerated backbone degradation and loss of sulfonic groups.

Effect of framework stabilization (sSLM, clays and carbon nanotubes): The use of rigid inorganic layered frameworks (sSLM) and carbon nanotubes fixed at interfacial boundaries makes it possible to dramatically reduce anisotropic membrane swelling (the swelling ratio decreases from 40–50% to an acceptable 12–15%). In relative humidity cycling (RH cycling) tests, such systems successfully exceed the threshold of 20,000 cycles without a critical increase in hydrogen crossover, retaining up to 85–90% of their initial conductivity after 500–1000 h of testing.

Table 1 summarizes recent Nafion- and PFSA-based composite membranes aimed at stabilizing proton conductivity under low relative humidity and elevated temperature. The selected studies show that the greatest improvements are usually achieved through a combination of water retention, additional proton-conducting sites, and stabilization of the ionomer/filler interface.

### 5.3. Regulation of the Ionomer’s Structure Through Various Pre-Treatment Techniques

The following methods of structure control are most commonly used to produce water-independent membranes [43,61,62]:

- Control of the ionomer structure by regulating RH during the drying process;
- Use of different solvents;
- Selection of ionomers with varying equivalent masses;
- Implementation of thermal treatment.

The primary aim of these pretreatment methods is to optimize the internal nanostructure of the ionomer (the size and connectivity of water channels) in order to ensure efficient proton conductivity even at low water content, thereby creating membranes whose performance shows minimal dependence on external humidity levels. This is critically important for applications in devices operating without external humidification (for example, in self-humidifying fuel cells).

#### 5.3.1. Controlled Drying Under Conditions of Low RH

When ionomer films are dried at low RH (for example, 0–20%), a denser and more ordered structure with smaller hydrophilic domains is formed. The applied treatment increases mechanical stability and limits swelling at elevated humidity levels, thereby reducing the membrane’s sensitivity to humidity variations during operation. 

The approach of structuring the membrane via drying at different humidity levels is described in the scientific literature. For example, the authors of [63] experimentally demonstrated that drying under very low humidity conditions (around 9% RH) forms an ultra-dense structure that effectively resists excessive swelling, prevents catalyst and ionomer leaching, and dramatically enhances mechanical stability (in contrast to drying at high humidity, which leads to chain mobility and mechanical instability). In [64], it was shown that fine-tuning of humidity during thermal treatment of PFSA membranes optimizes the structure and improves the balance between conductivity and mechanical stability.

It should be noted that the strategy of reducing hydrophilic domain size by drying at low RH may, at first glance, contradict the design principles of alternative hydrocarbon membranes (such as SPEEK), where researchers, on the contrary, actively seek to enlarge hydrophilic domains. However, this difference is fundamentally rooted in the difference in the initial microstructure of PFSA ionomers and aromatic hydrocarbon backbones. Nafion possesses a highly flexible fluorocarbon matrix that naturally provides strong microphase segregation, forming well-connected and broad ion channels approximately 4 nm in diameter [57]. Slight densification of these domains upon drying under low humidity conditions optimizes the trade-off between swelling resistance and proton flux without losing the connectivity of the conducting pathways [64].

In contrast, alternative aromatic matrices have a rigid hydrocarbon backbone with weak phase segregation, which initially leads to narrow (on the order of 1–2 nm), dead-end, and highly tortuous channels [65]. Consequently, for alternative hydrocarbon membranes, increasing domain size and enhancing their connectivity (often via the targeted introduction of special additives) is an obligatory path to approach the baseline percolation of Nafion [43]. Thus, domain size optimization addresses two fundamentally different challenges depending on the polymer type: stabilization of the already established conductive system in PFSA versus forced construction of channels in the rigid and constrained hydrocarbon matrix.

#### 5.3.2. Employing Different Solvents

The solvent type used in the ionomer dispersion significantly influences polymer chain self-assembly and the resulting morphology of the cast film. This occurs because the solvent’s physicochemical properties, particularly its dielectric constant, determine the balance between hydrophobic and hydrophilic interactions within the ionomer. Consequently, solvent selection enables control over aggregate size and distribution, as well as the ionomer film structure on the catalyst surface, thereby affecting proton conductivity, gas transport, and mechanical properties [66,67].

Experimental studies using dynamic light scattering have directly correlated solvent choice with aggregate size. Comparative analysis of ionomer dispersions revealed that N-methylpyrrolidone produces significantly smaller particles (~0.40 μm) than water–isopropanol mixtures (~2.02 μm) [68]. The membrane–electrode assembly prepared from dispersions with larger particle sizes (water–isopropanol) exhibited better initial performance, while those from smaller particle sizes (N-methylpyrrolidone) showed superior durability, demonstrating that solvent selection can be optimized based on specific application requirements [68].

One of the approaches to controlling polymer structure is the use of various dispersing media prior to membrane casting. Safronova et al. [69] examined dispersions of PFSA polymer Nafion in various dispersing liquids (water–isopropanol mixtures in different ratios, aprotic solvents such as N,N-dimethylformamide and N-methyl-2-pyrrolidone, ethylene glycol) and with different counterions. It has been shown that the polymer morphology in dispersion determines the mechanical properties of membranes cast from Nafion dispersions in the Li^+^ form. Membranes obtained from dispersions in aprotic solvents exhibited the highest strength and stiffness. High macromolecular mobility in dispersions with aprotic solvents and a low degree of aggregation promote effective macromolecule interconnection and the formation of films with good mechanical properties. Membranes prepared from isopropanol–water, dimethylformamide, and N-methyl-2-pyrrolidone solutions show elevated water absorption and proton conductivity. Membranes cast from dispersions in aprotic solvents developed small, uniformly distributed pores with high interconnectivity. As a result, the rate of non-selective transport through such membranes was lower compared to membranes prepared from isopropanol–water mixtures.

Solvent selection thus provides a powerful tool for tuning ionomer morphology across multiple length scales from molecular aggregation in solution [66] to nanoscale film structure [67,68] and macroscale transport properties [69]. The choice of dispersing medium should be guided by the desired balance between performance (proton conductivity, gas transport) and durability (mechanical stability, resistance to degradation).

#### 5.3.3. Modification of the Ionomer’s EW and Side-Chain Length

The use of ionomers with lower EW (e.g., Aquivion D79-25BS compared to Nafion) leads to stronger phase separation and the formation of well-defined hydrophilic and hydrophobic domains. Membranes with lower EW may exhibit better performance at low humidity, as they have a higher concentration of ion-exchange groups and an improved ability to retain water.

For example, in the study by Park et al. [61], various ionomer dispersions with different equivalent weights, side-chain lengths, and dispersing solvents were analyzed to investigate the influence of the microstructure of catalyst layers on MEA performance in PEMFCs. Five commercially available perfluorosulfonic acid-based ionomer dispersions were used to prepare laboratory propylene-glycol-based ionomer dispersions. The thickness of self-organized ultrathin films on a silicon wafer was measured for all types of ionomer dispersions, which mainly correlated with the type of dispersing solvent (water, 2-propanol, and propylene glycol), the ionomer side-chain length, and wettability. As a result, ionomer dispersions with short side chains and propylene-glycol-based dispersions formed thicker films due to the smaller average size of their ionomer aggregates. Catalyst layers with a thick ionomer coating, specifically prepared using propylene-glycol-based ionomer dispersions, did not affect activation losses but reduced ohmic losses by approximately 42% and increased the limiting current density by 43% in PEMFCs. Additionally, it was confirmed that slightly higher performance occurs with increasing ultrathin ionomer film thickness due to the influence of side-chain length. Catalyst layers with ionomer films thicker than 100 nm demonstrated higher performance than layers with thicknesses below 100 nm. MEAs employing Nafion D2020 with a hydrophobic surface exhibited a lower growth factor (2.26%) compared to those using Aquivion D98-25BS with a hydrophilic surface, due to differences in water retention.

#### 5.3.4. Application of Thermal Treatment

Thermal treatment of PFSA membranes increases their strength and affects their water uptake [70]. Increased crystallinity of the hydrophobic matrix prevents pore enlargement during hydration; therefore, the higher the treatment temperature, the lower the water uptake (the hydration number per functional group λ(H_2_O/–SO_3_H) is 35 and 25 for Nafion membranes with EW = 1100 in the proton form, cast at 130 and 190 °C, respectively [71]. A 10% decrease in methanol permeability was reported for thermally treated Nafion^®^ membranes [72]. Hydrothermal treatment of PFSA membranes, in contrast, results in increased water uptake and ionic conductivity as the treatment temperature rises [25,73,74]. PFSA membranes treated at temperatures close to the glass transition temperature exhibit softening and a lowered Young’s modulus due to a reduction in crystallinity [75].

Pretreatment of membranes aimed at creating a specific structure has demonstrated its effectiveness in improving and stabilizing membrane performance. However, given the dynamic structural behavior of Nafion-like membranes under operating conditions, concerns exist regarding degradation of the structure formed during pretreatment over long-term membrane operation.

Despite the obvious advantages of thermal treatment (such as annealing, hydrothermal aging, and high-temperature conditioning) in improving mechanical stability and reducing fuel crossover, a detailed quantitative analysis of the literature reveals a serious drawback: in the vast majority of cases, these procedures lead to a noticeable decrease in proton conductivity of the membranes [70,71]. This is the flip side of the coin—the price paid for improved mechanical and barrier properties. To objectively assess the feasibility of such strategies, it is necessary to examine in detail the underlying physicochemical mechanisms.

1. Molecular relaxation and shrinkage of hydrophilic channels

During annealing at temperatures above the ionomer glass transition temperature (T_g_~110–130 °C for Nafion), perfluorinated macromolecules acquire high conformational mobility.

Mechanism: The structure of the conducting channels, which in as-extruded or cast membranes is in a metastable (strained) state, begins to relax toward the thermodynamic free energy minimum [25].

Consequences: Densification of the polymer matrix occurs, accompanied by a decrease in free volume and shrinkage of the average diameters of hydrophilic ionic domains. As a result, the water sorption capacity of the membrane decreases: the hydration parameter λ irreversibly drops by 15–25%, leading to a 20–40% decrease in proton conductivity compared to untreated pristine samples [70].

2. Increase in crystallinity of the polymer backbone

Thermal exposure catalyzes the rearrangement of not only the hydrophilic phase but also the hydrophobic phase.

Mechanism: Prolonged holding of the membrane at elevated temperatures (especially in the range of 120–140 °C) stimulates local recrystallization of polytetrafluoroethylene segments of the main backbone [73].

Consequences: Increased crystallinity of the hydrophobic matrix rigidly fixes the polymer network. On the one hand, this provides a huge gain in mechanical strength and reduces swelling. On the other hand, crystalline regions act as steric barriers: they reduce the lateral mobility of sulfonic acid groups (–SO_3_H) and bend the diffusion pathways of hydrated protons (the tortuosity factor increases), leading to a decrease in the self-diffusion coefficient of water and protons [25,72].

3. Hydrothermal aging under pressure

Extreme treatment conditions in liquid water under elevated pressure and temperatures above 100 °C (hydrothermal treatment) lead to a specific morphological rearrangement.

Mechanism: Water under elevated pressure acts as a powerful plasticizer. Under the influence of temperature, avalanche-like microphase segregation occurs, during which ionic groups coalesce into isolated clusters [71,73].

Consequences: Although water is retained well locally within such clusters, the connectivity (percolation) between neighboring water domains is disrupted. The rupture of the unified transport network blocks the Grotthuss relay mechanism, reducing overall conductivity.

4. Peculiarities of thermal treatment of fluorine-free alternatives (SPEEK)

For alternative hydrocarbon membranes, such as SPEEK, thermal annealing or sol–gel crosslinking with inorganic agents (e.g., tetraethoxysilane, TEOS) is often a necessary measure; without it, the membrane dissolves in water at temperatures above 60 °C [74,75].

Mechanism and trade-off: Thermal fixation of the SPEEK structure severely restricts the conformational flexibility of the aromatic rings. As a result of rigid crosslinking or matrix densification, proton conductivity drops even more sharply than in Nafion; in some cases, the reduction reaches 50–60% of the initial potential [75].

Thus, the application of thermal treatments always represents a severe trade-off between transport and mechanical properties. The annealing strategy is fully justified for systems operating under high pressures or in DMFCs, where a 20–30% reduction in conductivity is more than compensated by a 50–70% decrease in methanol permeability [70] and an increase in mechanical durability. However, for classical hydrogen-fueled PEMFCs, harsh annealing regimes above 120 °C require strict moderation, as the resulting drop in proton flux may negate all the advantages gained from structural stabilization.

#### 5.3.5. Relationship Between Hydration State and Proton Conductivity

The effects of the pretreatment methods described above (Section 5.2.1 and Section 5.2.2) on proton conductivity can be understood in terms of the membrane hydration state. The fundamental relationship between the water-to-sulfonic acid ratio (λ) and proton conductivity, including the critical thresholds λ = 3 and λ = 6, is discussed in Section 5.1.1. Consequently, an extensive approach to stabilizing conductivity involves increasing the number (density) of water channels through tighter polymer chain packing. This has been successfully demonstrated in studies focused on modulating the membrane morphology via controlled drying conditions or the selection of specific casting solvents that alter the ionomer aggregate size. A secondary, less intuitive route involves increasing lambda by modifying the equivalent weight (EW) and tuning the length of the polymer side chains.

However, a critical analysis of the long-term durability of membranes modified via these matrix-tuning techniques reveals that their morphology is inherently labile. Under operational fuel cell conditions, characterized by dynamic hygrothermal cycling, these structures tend to undergo structural relaxation toward thermodynamically stable, less conductive states [57,76].

The repeated swelling and shrinkage induce significant internal mechanical stress, leading to irreversible macromolecular restructuring, localized domain collapse, and a subsequent decline in water retention capacity over extended operation [57]. Therefore, these matrix and solvent-tuning approaches should not be considered fully mature stabilization technologies unless these structural relaxation limitations are properly addressed.

### 5.4. Nafion Alternatives

A number of membranes have been developed as alternatives to Nafion, offering either improved humidity independence or advantages in other aspects (environmental impact, thermal stability, and others), which enable their operation at higher temperatures and/or under low RH conditions [75,77,78].

The primary goal of developing these alternatives is to enable efficient fuel cell operation at high temperatures (100 °C and above) without relying on the bulky external humidification systems necessary for Nafion.

The following sections describe the main alternative approaches.

#### 5.4.1. Sulfonated Aromatic Polymers

**Figure 2:**
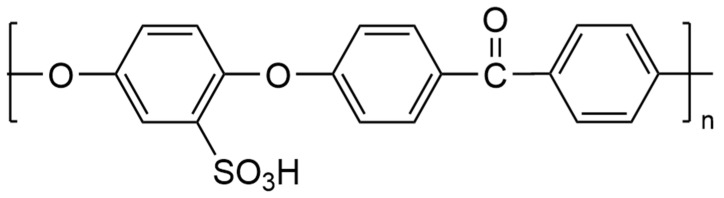
Structural formula of SPEEK.

SPEEKs (Figure 2), along with other sulfonated aromatic polymers, are reported to be among the most promising and cost-effective alternatives to Nafion [75]. Sulfonated aromatic polymers are an important class of materials that serve as effective substitutes for Nafion-based fuel cell membranes. These polymers consist of an aromatic polymer backbone and a proton-conducting polymer electrolyte. The aromatic polymers themselves are primarily composed of benzene rings or aromatic heterocyclic rings [54,57].

The connections between aromatic rings may be regular and rigid or irregular, resulting in branched and more flexible structures. This structural diversity allows sulfonated aromatic polymers to combine thermal stability with proton-conducting functionality, which is essential for fuel cell applications. Aromatic polymers can be sulfonated by treatment with concentrated acid solutions, including sulfuric acid, chlorosulfonic acid, pure or complexed sulfur trioxide, and acetyl sulfate [75]. After the introduction of sulfonic acid groups, thermally stable aromatic polymers acquire proton-conducting properties.

Although sulfonated aromatic polymers often exhibit lower absolute proton conductivity than Nafion under fully hydrated conditions, their conductivity may decrease less sharply with increasing temperature [79,80]. In this context, SPEEK is of particular interest because it generally demonstrates lower degradation, lower activation energy for proton transport, and better retention of proton conductivity at elevated temperatures. Due to the presence of densely packed sulfonic acid groups, SPEEK can maintain proton conductivity with relatively limited losses at high temperatures and may outperform Nafion at 100 °C. Therefore, SPEEK is considered a suitable alternative to Nafion for operation above 100 °C, where Nafion typically begins to lose efficiency.

These polymers demonstrate favorable proton conductivity and chemical durability and may be employed as low-cost alternatives to Nafion. Their properties may be optimized to reduce dependence on water. However, it should be noted that proton conductivity in SPEEK-based membranes is strongly affected by additional parameters, including relative humidity, ion-exchange capacity, and the degree of sulfonation. These factors are critical for evaluating the suitability of SPEEK as a replacement for Nafion in proton-exchange membranes and require systematic consideration.

Despite its advantages, the practical application of SPEEK is limited by the so-called “SPEEK trade-off”: at a high degree of sulfonation, typically above 60–70%, proton conductivity increases substantially, but this improvement is accompanied by excessive water uptake and swelling, reduced mechanical strength, and, in some cases, partial or complete dissolution of the membrane in hot water [81].

To overcome this limitation, significant progress has been achieved in the development of strategies aimed at stabilizing both the structure and proton conductivity of SPEEK-based membranes [75,82]. The main approaches are discussed below, with particular emphasis on suppressing excessive swelling, improving mechanical and chemical stability, and preserving efficient proton-conducting pathways.

1. Incorporation of 2D nanomaterials and carbon-based frameworks

One of the most widely explored strategies is the incorporation of functionalized two-dimensional nanomaterials and carbon-based fillers into the SPEEK matrix. Such additives can form strong hydrogen-bonding interactions with the sulfonic acid groups (–SO_3_H) of SPEEK, thereby promoting the organization of proton-conducting channels, restricting excessive polymer-chain mobility, and suppressing membrane swelling [83].

Porous graphitic carbon nitride (Pg-C_3_N_4_) has been shown to be an effective nanofiller for simultaneously improving proton conductivity and mechanical stability. The incorporation of only 3 wt.% Pg-C_3_N_4_ nanosheets into the SPEEK matrix resulted in an optimized proton conductivity of 138 mS·cm^−1^ at 80 °C and 100% relative humidity. At the same time, the tensile strength increased by approximately 50%, reaching 74.1 MPa. This improvement was attributed to interactions between the nitrogen-containing groups of the filler and the acidic groups of the SPEEK matrix.

Fluorinated graphite (FG) is another promising carbon-based modifier. SPEEK/FG composite membranes demonstrate enhanced oxidative, thermal, and mechanical stability compared with pristine SPEEK. The formation of hydrophobic–hydrophilic interfacial regions helps reconstruct proton-transport pathways while maintaining the swelling ratio at a relatively stable level of 29.4%. In [83], the authors described a composite SPEEK membrane reinforced with fluorinated graphite (SPEEK/FGx), which overcame the typical stability limitations of highly sulfonated SPEEK while significantly improving tensile strength, oxidative stability, thermal stability, and power density. In particular, compared with pristine SPEEK, the SPEEK/FG0.5 membrane achieved a 175% improvement in oxidative stability and a swelling ratio of only 29.4%, leading to a 6.8% increase in the maximum power density of a single-cell PEMFC. Experimental and theoretical analyses showed that the enhanced performance resulted from the synergistic interaction between the newly formed hydrogen-bonding network and the hydrophobic FG interface, which jointly optimized the polymer-chain structure and reconstructed efficient proton-transfer pathways.

Magnetically oriented graphene oxide represents another effective approach to improving proton transport, especially under low-humidity and high-temperature conditions. The use of functionalized graphene oxide containing Fe_3_O_4_ particles oriented under an external magnetic field enables the formation of directional proton-conducting channels. As a result, the membrane reached a proton conductivity of 11.13 mS·cm^−1^ at 120 °C and 20% RH, exceeding the value reported for commercial Nafion under the same conditions, 9.78 mS·cm^−1^ [84].

2. Reinforcement with nanofibrous frameworks

Another strategy for suppressing macroscopic swelling in highly sulfonated SPEEK membranes without blocking active proton-transport sites is the transition from dispersed fillers to continuous one-dimensional nanofibrous frameworks. In such composite systems, SPEEK acts as the proton-conducting matrix, whereas the polymer nanofiber network provides mechanical reinforcement and dimensional stabilization.

A representative example is a trilayer PI/SPEEK/PI membrane, in which an inexpensive proton-conducting SPEEK matrix is sandwiched between electrospun polyimide (PI) nanofiber layers [85]. This rigid nanofibrous scaffold protects the polymer matrix from operational mechanical and hydrolytic stresses, resulting in a pronounced improvement in hydrolytic and dimensional stability. Importantly, the PI/SPEEK/PI membrane retains an initial proton conductivity comparable to that of pristine SPEEK but demonstrates substantially improved durability. During six-week accelerated stress tests, the conductivity loss of the PI/SPEEK/PI membrane was only 21%, which was significantly lower than that of commercial Nafion 117 (30%) and pristine SPEEK (55%). FTIR, XPS, and TEM analyses confirmed that intermolecular hydrogen bonding at the PI/SPEEK interface induces the self-organization of a dense interfacial layer enriched with sulfonic acid groups, which contributes to stable and rapid charge transport [85].

A related reinforcement strategy involves the incorporation of liquid-crystal-modified aramid fibers into the SPEEK matrix. For example, a composite membrane containing 1 wt.% liquid-crystal-modified aramid fibers (DBAF) reached a proton conductivity of 230 mS·cm^−1^ at 80 °C, representing a 64% increase compared with pristine SPEEK [86]. This result further confirms that continuous or semi-continuous reinforcing frameworks can improve proton transport while contributing to mechanical stabilization.

3. Three-dimensional covalent and ionic cross-linking

Cross-linking is an effective strategy for improving the dimensional and hydrolytic stability of SPEEK membranes, particularly at high degrees of sulfonation. The formation of a three-dimensional network restricts the mobility of polymer chains and prevents structural degradation or dissolution in hot or boiling water, even at very high DS values [87].

Thermal cross-linking with polyhydric alcohols, such as ethylene glycol or glycerol, leads to the formation of stable ester bridges between sulfonic acid groups. These cross-linked membranes exhibit improved hydrolytic stability and mechanical strength, while maintaining the continuity of proton-conducting channels. As a result, their stability can approach that of Nafion 117, while preserving the intrinsic advantages of the SPEEK matrix.

Ionic polymer blending is another approach to stabilizing SPEEK-based membranes. In this case, SPEEK is mixed with basic or rigid-chain polymers to reduce swelling and improve ion selectivity. For example, a composite membrane based on sulfonated polyphenylene sulfide (SPPS) and SPEEK containing 15 wt.% SPPS demonstrated an ion selectivity of 10.5 × 10^3^ S·min·cm^−3^, which is approximately 2.5 times higher than that of Nafion 117, 4 × 10^3^ S·min·cm^−3^, and enabled an energy efficiency of up to 90% in vanadium redox flow battery tests. SPEEK/SPPO blends, where SPPO denotes sulfonated polyphenylene oxide, have also been actively investigated as a route to improving the mechanical and dimensional stability of proton-exchange membranes [88,89].

In addition, the development of composite membranes with ionic liquids has shown promise. A SPEEK/IL/GO-2% composite achieved a proton conductivity of 21.35 mS·cm^−1^ at 120 °C, with ionic liquid losses of only 20.17% due to physical crosslinking [90]. More recently, Lv et al. [91] developed an ionic–covalently cross-linked SPEEK membrane (C-SPEEK/IL/GO-1%) that achieved a proton conductivity of 47.43 mS·cm^−1^ at 120 °C while maintaining excellent dimensional stability. The large hydrogen bond network between the ionic liquid, graphene oxide, and SPEEK provides additional sites for proton hopping via the Grotthuss mechanism [90,91].

4. Self-organization of ionic channels using morphology-inducing agents

Directed self-organization of ionic clusters is a promising approach to enhancing proton conductivity in SPEEK membranes without necessarily increasing the degree of sulfonation. In this strategy, low-molecular-weight agents are used to reorganize the morphology of the SPEEK matrix and enlarge the ionic domains responsible for proton transport.

For example, the introduction of n-butanol (n-BuOH) as a self-organization agent allows the size of ionic clusters in SPEEK to increase from approximately 1 nm to 3 nm through directed hydrogen-bonding interactions. The resulting membrane achieved a high proton conductivity of 314 mS·cm^−1^ at 80 °C, which is 88% higher than that of Nafion 115. Moreover, this advantage was maintained over a broad range of relative humidity, from 40% to 90% RH [92].

Recent advances in molecular design have also demonstrated that grafting flexible sulfonic acid-containing side groups onto SPEEK can significantly improve proton transport. For instance, Yu et al. developed a fully grafted SPEEK membrane containing multiple flexible propanesulfonic acid groups (MS-SPEEK-102), which achieved a proton conductivity of 83.0 mS·cm^−1^ at 80 °C and 98% relative humidity, along with a power density of 0.530 W·cm^−2^ at 60 °C [93]. Furthermore, a composite membrane incorporating 1 wt.% liquid-crystal-modified aramid fibers (DBAF) into the SPEEK matrix reached a proton conductivity of 230 mS·cm^−1^ at 80 °C, representing a 64% increase over pristine SPEEK [86]. This approach illustrates that not only filler incorporation but also targeted modification of the SPEEK molecular architecture can enhance the connectivity of proton-conducting domains.

5. Encapsulation of ionic liquids in MOF frameworks

The incorporation of metal–organic frameworks containing ionic liquids within their pores represents another strategy for improving SPEEK-based membranes. Such IL@MOF systems, for example, those based on UiO-66 and the ionic liquid TEA-PS.HSO_4_, help address the problem of dopant leaching from the membrane while enhancing proton conductivity. When 7.5 wt.% of this filler was introduced into SPEEK, the proton conductivity remained stable in the range of 92–140 mS·cm^−1^. In addition, the composite structure limited excessive water sorption, thereby improving the dimensional stability of the membrane [94].

6. Other composite sulfonated polymer membranes

Alongside SPEEK-based systems, other sulfonated polymer composite membranes have also been investigated as potential alternatives to Nafion. Al-Mashhadani et al. [78] studied composite membranes based on a blend of sulfonated poly(vinyl alcohol) and Pebax-1657 with the addition of TiO_2_PO_4_ nanoparticles. An optimal filler loading of 3 wt.% provided maximum water uptake and ion-exchange capacity. In fuel cell tests performed at 80 °C and 80% RH, the membrane exhibited a power density of 62 mW·cm^−2^. However, the long-term stability of these membranes remained beyond the scope of that study.

To systematize the approaches discussed above, Table 2 summarizes the key achievements, advantages, and remaining technical limitations of the main stabilization strategies for SPEEK-based membranes.

Thus, modified SPEEK membranes are no longer regarded only as a low-cost but compromise-based alternative to fluorinated polymers. Recent advances in nanostructuring, nanofibrous reinforcement, covalent and ionic cross-linking, morphology-induced self-organization, and hybrid filler design have substantially improved the balance between proton conductivity and membrane stability. Under specific operating conditions, optimized SPEEK-based membranes can outperform Nafion-type membranes in terms of peak proton conductivity, initial mechanical resistance, dimensional stability, or reduced ion/fuel crossover. However, these advantages are strongly dependent on membrane composition, degree of sulfonation, relative humidity, operating temperature, and test protocol. Therefore, despite significant laboratory-scale progress, the long-term chemical and operational durability of SPEEK-based membranes under realistic dynamic fuel-cell conditions remains an open issue and requires further systematic investigation.

#### 5.4.2. Polybenzimidazole (PBI) Membranes

Polybenzimidazole (PBI) and SPEEK are materials stable at high temperatures (up to 200 °C). To achieve proton conductivity, substantial acid doping is required. The acid must satisfy the requirements of thermal stability at operating temperatures and structural stability within the material (it must not be washed out of the membrane during operation). The literature describes both pristine membranes and composite membranes containing a variety of nanostructured fillers. Phosphoric acid and its derivatives, for example, phosphotungstic heteropolyacids (HPAs), are most commonly used as doping acids.

Sun et al. [14] reported that acid-doped PBI membranes demonstrate excellent conductivity under high-temperature and low-humidity conditions, along with favorable power density. However, most systems function well above 150 °C, and there are no documented studies at lower operating temperatures approaching the U.S. Department of Energy (DOE) target of 120 °C. It has been shown that the addition of zirconium phosphate enhances the conductivity of PBI membranes at temperatures up to 200 °C, but the detailed mechanism of interaction between this filler and the PBI matrix is unclear, and the stability of this composite membrane at such high temperatures has been hardly studied. It is reasonable to assume that the high conductivity is associated with phosphoric acid formed during decomposition at high temperature.

Composite membranes containing phosphotungstic HPA and their modifications (Cs_2.5_H_0.5_PW_12_O_40_; H_3_PW_12_O_40_/SiO_2_) demonstrate high proton conductivity under anhydrous conditions at 150 °C, attributed to HPA, providing additional surface functional sites through the composite membrane to facilitate proton transport, since protons are transferred to the HPA surface. The ESF-BP/7PWS composite membrane exhibits a proton conductivity of 0.20 S·cm^−1^ at 95 °C and 100% RH [95].

Data regarding the properties of PBI membranes suitable for relatively high-temperature applications are reported [55,59]. It is highlighted that for a highly efficient polymer electrolyte, stable mechanical properties and high proton conductivity on the order of 10^−2^ S·cm^−1^ under low-humidity (or anhydrous) and high-temperature conditions are ultimately required. Emphasizing this practical principle, doped PBIs have become one of the leading materials as proton-conducting membranes, exhibiting high proton conductivity with values up to 10^−2^ S·cm^−1^.

The structural versatility of these membranes, including the introduction of sulfonic groups at different positions within the PBI molecule and the use of diverse acidic groups (–SO_3_H; –PO_3_H_2_), enables the variation in key properties such as proton conductivity, mechanical strength, and thermal stability. This approach provides the possibility of flexible tuning of the functional characteristics of the membranes. The operational temperature of these membranes reaches up to 200 °C.

Among the various types of membranes studied, phosphoric acid-doped PBI membranes show comparable performance that meets most of the DOE targets (except durability) for HT-PEM. The modification of HT-PEM based on PBI should be focused on how to improve durability. Achieving a balance between proton conductivity and mechanical strength, while limiting phosphoric acid loss during fuel cell operation, remains a key challenge for PBI-based HT-PEM development.

#### 5.4.3. Critical Analysis of the Durability of Alternative Fluorine-Free and High-Temperature Membranes

Examination of alternative classes of polymer matrices, SPEEK, sulfonated poly(arylene ether)s (SPA), PBI, and fluorine-free blend systems, reveals specific degradation trade-offs that differ from those of classical PFSA membranes (Nafion). The main challenge for such systems lies in coupling the thermal stability of aromatic backbones with hydrolytic and chemical stability during long-term operation.

1. Degradation trade-off of aromatic polymers (SPEEK and SPA)

The use of SPEEK and sulfonated poly(arylene ether)s is considered a cost-effective and environmentally friendly hydrocarbon alternative to Nafion [75]. However, their durability is strictly limited by the degree of sulfonation [65].

Degradation mechanism: Achieving high proton conductivity requires a high DS, which irreversibly leads to excessive swelling, loss of mechanical properties, and dissolution of the polymer in water at elevated temperatures [65]. Under AST humidity cycling, the labile channel structure of hydrocarbon polymers undergoes severe viscoplastic deformation [75].

Chemical vulnerability: Unlike the perfluorinated backbone of Nafion, the aromatic rings and ether linkages of SPEEK and SPA are more vulnerable to nucleophilic attack by •OH and •OOH radicals. This leads to accelerated chain degradation, loss of sulfonic groups, and a sharp reduction in membrane lifetime during OCV tests [65].

2. Stabilization of fluorine-free blends with nanoparticles (S-PVA/PEBAX)

Innovative fluorine-free membranes based on blends of sulfonated poly(vinyl alcohol) (S-PVA) and polyether-block-amide (PEBAX 1657) are of great interest for intermediate-temperature applications but require rigid structural fixation [78].

Role of nanomodifiers: The introduction of titanium dioxide phosphate (TiO_2_PO_4_) inorganic nanoparticles acts as a powerful factor for spatial stabilization of the matrix [78]. Phosphated nanoparticles not only retain strongly bound water (increasing λ) but also act as physical crosslinking nodes.

Impact on durability: The presence of extended interfacial interaction between the S-PVA/PEBAX polymer chains and the TiO_2_PO_4_ surface effectively suppresses macromolecular relaxation and inhibits swelling of the hydrophilic phase [14]. This prevents membrane cracking and preserves the continuity of proton-conducting pathways under hydrothermal stress.

3. HT-PEM and the stability problem of heteropolyacids

The transition to the high-temperature range (120–200 °C) requires the use of composite materials with thermally stable dopants [14]. The introduction of heteropolyacids (HPAs) into sulfonated aromatic matrices (SPA) is aimed at maintaining conductivity under low-humidity conditions [95].

Critical durability defect: HPAs have high solubility in water. Under medium- and high-temperature operating conditions accompanied by periodic moisture condensation (e.g., during start-stop cycles), intensive leaching of HPAs from the SPA matrix occurs [14,95].

Consequences: Leaching of catalytically active dopants results in an irreversible drop in conductivity. Moreover, migration of HPAs to the electrodes causes poisoning of the platinum catalyst, sharply reducing the overall service life of the entire fuel cell assembly [95].

4. PBI matrices and phosphoric acid retention

Polybenzimidazole membranes doped with phosphoric acid (H_3_PO_4_) are the technological basis for HT-PEM systems operating without external humidification [77,96]. Their durability is determined by the strength of acid retention within the polymer network.

Degradation mechanism: Phosphoric acid is retained in PBI through hydrogen bonding and proton transfer to imidazole rings. However, during long-term operation, electro-osmotic drag and water vapor generated at the cathode cause gradual loss (“sweating out” and leaching) of free acid [77].

Mechanical fatigue: Acid loss leads to severe shrinkage of the PBI matrix, loss of plasticity, and a sharp drop in conductivity. Thermal cycling causes accumulation of internal stresses at membrane–electrode interfaces, leading to delamination and gas crossover [96]. A promising solution for improving the durability of such systems is covalent crosslinking or the creation of organo-inorganic networks that rigidly trap acid molecules within the pore volume [77,96].

Thus, while for PFSA membranes the key durability issues are viscoplastic fatigue and radical degradation, for alternative aromatic and fluorine-free systems the dominant challenges are the kinetic instability of dopants (leaching of HPAs and phosphoric acid) [14,77] and the need to strictly suppress the conformational lability of hydrocarbon polymer macromolecules [65,78].

Despite the excellent proton conductivity of phosphoric acid-doped polybenzimidazole (PBI) membranes at elevated temperatures, their widespread commercial adoption remains hindered by long-term stability issues, primarily arising from gradual acid leaching and a concomitant decline in mechanical strength. A promising solution to this challenge is the nanostructural modification of PBI matrices using porous aromatic frameworks (PAFs) [97]. As recently demonstrated by Spasov et al., the incorporation of amino-functionalized PAFs induces the formation of an extensive hydrogen-bond network between the functional groups of the additive and the polymer backbone. This not only effectively immobilizes phosphoric acid within the pores, minimizing its loss during operation, but also markedly improves the transport and mechanical properties of the composite under demanding thermal conditions. Consequently, the design of PBI membranes incorporating PAF architectures represents a promising direction in the development of alternative materials for high-temperature fuel cells.

#### 5.4.4. Composite Membranes with a Non-Nafion Matrix

The incorporation of additives into a non-Nafion matrix serves both purposes shared with Nafion-like matrices and specific ones. The general purpose is to ensure water retention under low humidity and relatively elevated temperatures. This problem is relevant for low-temperature fuel cells and for polymer matrix materials with comparatively limited thermal stability (such as polyvinyl alcohol (PVA), polyvinylidene fluoride (PVDF), hexafluoropropylene (HFP), and others). A specific purpose is to provide the membrane with proton conductivity. It is generally achieved through the addition of proton-exchange-capable materials. For example, a PVA matrix does not conduct protons and therefore requires modification with proton-conducting materials [98]. For this purpose, additives such as SPEEK and GO are used [99,100]. Furthermore, the use of PVA is associated with challenges related to optimizing long-term stability, improving contamination resistance, and increasing process scalability.

The use of PVDF and HFP is also associated with the need to add proton-exchange agents [101]. Proton-conducting properties are imparted through the addition of SPEEK.

Examples of improving the humidity independence of non-Nafion membranes are presented in studies [26,27]. Chen et al. [30] investigated a series of composite membranes with a non-Nafion matrix based on sulfonated poly(arylene ether sulfone) and GO (SPAES-GO-x). The properties of SPAES-GO-x membranes were evaluated, including proton conductivity, water uptake, swelling, and thermal stability. The incorporation of GO into the SPAES matrix suppressed swelling of the composite membranes and increased their proton conductivity. The SPAES-GO-3% membrane exhibited a conductivity value of 0.183 S·cm^−1^ at 120 °C and 100% RH. Compared with the pristine SPAES membrane, the swelling ratio of the SPAES-GO-2% membrane was reduced by 55.7% at 90 °C. SAXS showed that GO promoted the rearrangement of SPAES molecular chains to form larger ionic clusters, which enhanced proton transport.

Kim et al. [31] reported non-Nafion-type composite membranes based on sulfonated fluorinated multiblock copolymer (SFMC), SPEEK, and 1 or 5 wt.% GO. The membranes were prepared by solution casting. The hydrophilic–hydrophobic–hydrophilic structure of SFMC enhances compatibility with SPEEK and GO. A well-optimized ternary hybrid membrane containing 5 wt.% GO exhibited a maximum proton conductivity of 111.9 mS·cm^−1^, which is two times higher than that of the pristine SFMC membrane. The maximum PEMFC power density of 528.07 mW·cm^−2^ was obtained for the ternary hybrid membrane at a current density of 1321.1 mA·cm^−2^ when the cell was operated at 70 °C and 100% RH. For comparison, the pristine SFMC membrane demonstrated a maximum power density of only 182.06 mW·cm^−2^ at a current density of 455.56 mA·cm^−2^ under the same operating conditions.

It should be noted that Nafion, due to its specific properties (fluorocarbon backbone, high proton conductivity, high chemical and thermal stability, low gas permeability, ability to form monolithic dense films, good resistance to leaching by water and methanol, and others), remains the best choice to date.

#### 5.4.5. Critical Analysis of the Durability of Blend and Nanocomposite Membranes Based on PVA, SPEEK and Fluoropolymers

Investigation of blend polymer systems based on hydrophilic poly(vinyl alcohol) (PVA) and rigid-chain SPEEK in combination with nanofillers and fluoropolymer matrices (PVdF-HFP) makes it possible to solve the problem of excessive swelling of hydrocarbon membranes. However, quantitative analysis of their life cycle reveals specific degradation limitations associated with the chemical degradation of PVA and the stability of the formed semi-interpenetrating networks (IPNs).

1. Stabilization and degradation of PVA matrices: crosslinking and predictive modeling

The use of PVA as a base matrix is attractive due to its high density of hydrophilic functional groups and processability, which is actively supported by modern predictive modeling methods [98].

Durability and hydrolysis problem: Pure PVA is water-soluble and subject to rapid hydrolysis. Without chemical crosslinking (e.g., with glutaraldehyde), membranes disintegrate within the first 10–24 h of operation in a humid environment. Even crosslinked PVA systems at temperatures above 60–80 °C exhibit a gradual decrease in mechanical strength of 20–35% after 100–150 h of operation due to the breaking of labile ester bridges.

2. SPEEK/graphene oxide (GO) composites and electrospinning

The transition to membrane architectures obtained by electrospinning with the addition of graphene oxide (SPEEK/GO) makes it possible to significantly increase both initial conductivity and mechanical properties [100].

Quantitative effect of GO: The introduction of functionalized GO in an amount of 0.5–1.5 wt.% creates two-dimensional barriers that reduce the anisotropic swelling ratio of SPEEK from critical values of 60–80% to technologically acceptable values of 18–25%.

Mechanical fatigue limit: Strong hydrogen and covalent bonds between SPEEK sulfonic groups and oxygen-containing GO groups ensure structural retention. Under mechanical fatigue tests, such membranes can withstand up to 5000–7000 humidity cycles. However, during long-term tests (more than 300–500 h), local accumulation of radicals at defects in the GO carbon lattice can provoke accelerated chemical degradation (chain “unzipping” of the matrix), leading to an increase in hydrogen crossover.

3. Three-component blend systems with crosslinking agents (SPEEK/PVA/TEOS)

The integration of tetraethoxysilane (TEOS) as an inorganic crosslinking agent into SPEEK/PVA blends is aimed at simultaneously fixing both polymer phases [99].

Effect of inorganic network: During the sol–gel synthesis process, TEOS forms a rigid internal SiO_2_ network, which acts as a physical and chemical framework. This prevents phase separation (microphase segregation drift) between SPEEK and PVA.

Stability indicators: The presence of the SiO_2_ framework reduces the rate of conductivity degradation at elevated temperatures: the performance drop is no more than 10–15% after 200 h of testing (while uncrosslinked blends lose up to 50–60% of conductivity due to the destruction of conducting pathways).

4. Hydrophobic–hydrophilic balance in three-component systems (SPEEK/PVdF-HFP/SiO_2_)

The use of poly(vinylidene fluoride-co-hexafluoropropylene) (PVdF-HFP) copolymer as a hydrophobic component in a blend with SPEEK and SiO_2_ nanoparticles makes it possible to radically change the durability profile [101].

Mechanical and chemical protection: PVdF-HFP forms a chemically inert, fluorinated “skeleton” of the membrane that effectively protects the labile aromatic rings of SPEEK from attack by Fenton radicals. The SiO_2_ nanoparticles are localized in the hydrophilic domains, increasing the λ parameter.

Quantitative durability metrics: Such three-component systems demonstrate high stability in microbial fuel cells (MFCs) and PEM systems, successfully maintaining mechanical integrity (retaining 80–85% of the original tensile strength) over long-term durability tests lasting more than 500–1000 h. The leaching rate of the SiO_2_ additive is reduced by a factor of 3–4 compared to binary Nafion/SiO_2_ composites due to its effective entanglement at the SPEEK/fluoropolymer phase interface [30,31].

Thus, quantitative data show that binary hydrocarbon blends (SPEEK/PVA) without deep chemical modification have a limited operating life (up to 150–200 h). In contrast, the transition to three-component nanocomposite architectures (involving PVdF-HFP fluoropolymers, graphene oxide, or TEOS sol–gel frameworks) makes it possible to raise the mechanical and chemical endurance limit of the systems to 500–1000 h, preventing additive leaching and phase separation.

#### 5.4.6. Quantitative Durability Assessments of Alternative Membranes [14,65,75,77,78,95,96]

**Degradation trade-off of SPEEK and SPA:** To achieve conductivity comparable to Nafion (~0.1 S·cm^−1^), hydrocarbon membranes require a high degree of sulfonation (DS > 60–70%). However, at temperatures above 80 °C, this leads to catastrophic volumetric swelling (more than 80–100%) and mechanical destruction of the polymer matrix.

**Stabilization of fluorine-free blends (S-PVA/PEBAX) with TiO_2_PO_4_ nanoparticles:** The incorporation of phosphated titanium dioxide nanoparticles into the fluorine-free blend matrix makes it possible to reduce the swelling degree of the plastic PEBAX phase by a factor of 2–2.5, while simultaneously retaining strongly bound water at a level of λ ≥ 6 even at reduced relative humidity. Such nanocomposites demonstrate stable operation for 300+ hours without an avalanche-like drop in current characteristics.

**Dopant loss in HT-PEM (PBI/H_3_PO_4_) systems:** For high-temperature PBI membranes, the initial level of phosphoric acid doping is typically 200–300 mol.% (or a doping coefficient X_acid ~ 10–15 acid molecules per polymer repeating unit). The presence of water vapor at the cathode under operating temperatures of 160–180 °C leads to the leaching and loss of up to 20–40% of free (chemically unbound) acid during the first 500 h of operation.

**Lifetime limitations of HT-PEM:** Acid loss causes shrinkage of the membrane pore space and a drop in conductivity of 40–60%. The fuel cell voltage degradation rate increases from the target 2–5 μV·h^−1^ (for commercial standards) to an unacceptable 20–50 μV·h^−1^. This proves the need to move from simple doping to covalent crosslinking or the creation of robust organo-inorganic entanglement networks.

Table 3 summarizes recent studies on Nafion alternatives, including PBI-based HT-PEMs, sulfonated aromatic polymers, PVDF-derived membranes, and eco-friendly non-fluorinated systems. These materials are important because they address the limitations of PFSA membranes from different angles: high-temperature operation, lower cost, reduced fluorine content, and environmental sustainability.

## 6. The Most Promising Approaches to Stabilization

Among the approaches to stabilizing proton conductivity described above, three directions used in the development of nanocomposite membranes appear to be the most promising. These include the creation of oriented structures of proton-conducting channels, the incorporation of graphene-based materials into the membrane, and the introduction of MOFs. Why are these directions promising? Because they enhance the methods used to form composite membranes. Ordered and aligned proton-conducting channel structures within the membrane, all other factors being equal and given the advantages of the incorporated additive, enable control over the tortuosity of the proton transport pathway. A reduction in tortuosity enhances the effectiveness of the additive and increases proton conductivity. Graphene-based materials represent a unique group of materials with a readily controllable pseudo-2D structure and surface functional group composition. Such features provide increased flexibility in composite membrane technology. In addition, graphene is permeable to protons and impermeable to most other substances, including gases, fuel, and oxidant. Its incorporation greatly reduces reactant crossover. MOFs, due to their unique (lattice) structure, diversity, and ease of compositional tuning, allow targeted influence on the structure and properties of the membrane. These three approaches possess significant unexplored scientific, technological, and practical potential for improving membrane performance. These approaches address the fine-tuning of composition and structure at the supramolecular level, aiming to achieve materials with predetermined characteristics. Based on fundamental principles (formation of oriented structures and the incorporation of two-dimensional layers or framework architectures), these approaches enable variation in additive composition, size, and chemical properties, and consequently, membrane characteristics.

### 6.1. Oriented Structures and Composites, Including Those with Magnetic Properties

A notable recent trend is the use of ordered, uniformly distributed (regular) structures of moisture-retaining fillers to improve the water independence of proton exchange membranes.

In some studies, regular structures of ionomer and catalyst support are successfully used to increase the efficiency of transport processes. For example, Islam et al. [106] demonstrated that uniformly distributed nitrogen-containing functional surface groups on a spherical carbon support promote high ionomer coverage, which is directly confirmed by high-resolution electron microscopy and an electrical double-layer (EDL) capacitance that is nearly independent of humidity. The hydrophilic nature of the carbon surface appears to provide high activity and performance over a wide range of RH. The authors suggested that this effect is associated with the interaction between the ionic part of the ionomer and the nitrogen-containing functional group of the catalyst support. The carbon support consists of spherical particles with a diameter of approximately 200 nm.

Nicotera et al. [107] described a system of CNTs distributed within the membrane with titanium oxide nanoparticles deposited on them. This approach enabled the preservation of an effective nanodispersion of titanium oxide particles in the supporting matrix, thereby improving dimensional stability, hydrophilicity, and physicochemical properties of Nafion/multi-walled CNT–TiO_2_ (NMT-x) nanocomposites compared to pristine Nafion. At an optimal concentration (3 wt.% relative to the polymer), the nanocomposite membrane exhibited high transport properties with a strong water retention ability, resulting in proton conductivity of 8.3 mS·cm^−1^ at 80 °C and 20% RH. Titanium nanoparticles play a key role in retaining water molecules even under dehydrated conditions and also directly contribute to proton transport. In addition, long CNTs facilitate the formation of additional pathways for proton conductivity. These combined properties enabled the NMT-3 membrane to achieve a maximum power output of 307.7 mW·cm^−2^ in a single H_2_/air fuel cell (active electrode area of 5 cm^2^ and 0.5 mg Pt·cm^−2^ on both electrodes) under very harsh conditions, in particular at 120 °C and 30% RH. This result represents substantial progress in overcoming the limitations of conventional Nafion membranes. A key point in this system is the formation of a uniform filler distribution.

Controlled imparting of magnetic properties to Nafion enables the creation of materials with improved characteristics for various applications [108].

Proton-exchange membranes: composites with oriented magnetic nanoparticles provide higher proton conductivity and improved selectivity, which is important for fuel cells.

- Sensors: Magnetic properties may be used to create new types of chemical and biological micro- and nanosensors, particularly in systems requiring controlled material structuring.

Examples of such systems include the following composites with particles oriented in a magnetic field:

- γ–Fe_2_O_3_ (maghemite) nanoparticles. When introduced into Nafion, these nanoparticles can be oriented by an external magnetic field. This enables the formation of membranes with a directed microstructure. The alignment of the particles promotes the formation of ordered proton-conducting channels, thereby increasing ionic conductivity.
- Fe_3_O_4_ (magnetite) nanoparticles and CNTs. The creation of Nafion-based composites with magnetite nanoparticles and CNTs enables the concentration of magnetic particles in a specific region of the membrane under the influence of a magnetic field. Such structuring contributes to improved mechanical properties and the formation of functional layers useful in fuel cells.
- Paramagnetic ions (Fe^2+^ and Co^2+^). Doping Nafion with paramagnetic metal ions leads to the formation of hybrid systems exhibiting interesting magnetic and optical properties. For example, spin-crossover phenomena may be observed, which are amenable to investigation by NMR.

It should be noted that Nafion is a diamagnetic polymer. However, its unique structure and ability to form nanoscale water channels make it an ideal matrix for the creation of functional composites with induced magnetic properties, which may be useful in various advanced technologies and, in particular, for increasing the humidity independence of proton-exchange membranes.

In a comprehensive review on nanostructured proton-conductive polymer films [109], various strategies for achieving high proton conductivity through structural organization and molecular orientation are discussed and can be summarized as follows.

Key approaches include: (a) self-organized structures of PS-block-P4VP(MSA)_1.0_(PDP)_10_ [110]; (b) 2D SAXS profiles and measurements of in-plane and normal-to-plane conductivity of as-cast and aligned samples, demonstrating anisotropic proton conduction [111]; (c) multilayer films of poly(N-dodecylacrylamide-co-acrylic acid) prepared by the Langmuir–Blodgett method, exhibiting highly anisotropic proton conduction [112]; (d) a model showing the distance between acidic groups for high proton conduction [113]; (e) enhancement of proton conduction by molecular orientation of proton-conductive polymers; (f) aligned electrospun nanofibers of sulfonated polyimide [114]; (g) the structure of Nafion with an equivalent weight of 1100 (x = 6–7); and (h) a magnetically aligned composite membrane with proton transport in aligned channels, where PWA (phosphotungstic acid), CP4VP (ferrocyanide-coordinated poly(4-vinylpyridine)) act as electron-donating, proton-conducting, and redox polymer, and PSf (polysulfone) serves as a non-conductive polymer [115].

At present (as of late 2024–2025), there are no publications in the open scientific literature describing the formation of magnetic-field-oriented channels in Nafion containing C_70_ fullerene molecules.

Studies published in 2025 addressing Nafion composites with fullerenes or magnetic orientation in Nafion report the following [109]:

- Nafion/C_70_ composites. C_70_ molecules (as well as C_60_) are used in Nafion to modify the membrane morphology and improve properties, for example, to increase gas separation selectivity or proton conductivity. However, they are typically present in the form of agglomerates and are not oriented by a magnetic field.
- Magnetic orientation of channels in Nafion. This approach involves the introduction of anisotropic fillers (for example, CNTs, possessing distinct morphology and magnetic susceptibility) and aligning them with a strong external magnetic field during membrane formation. As a result, oriented (anisotropic) ion channels are formed, leading to increased proton conductivity in the direction of alignment.

Nonetheless, the spherical or near-spherical geometry of fullerene molecules does not confer sufficient magnetic anisotropy to enable their effective alignment under an external magnetic field within the Nafion matrix. Achieving anisotropic properties therefore requires fillers with pronounced geometric anisotropy (rods, tubes, plate-like structures, and others) or fillers functionalized with magnetic nanoparticles.

The fabrication of membranes with an ordered, directed channel structure, including via magnetic additives, is an effective and promising approach for stabilizing proton conductivity in membranes. At the same time, the use of magnetic materials capable of generating metal cations creates a risk of deterioration of the proton-conducting properties of the ionomer matrix as a result of ion exchange of protons for metal cations, especially in the long term.

### 6.2. The Application of Graphene Materials

**Figure 3:**
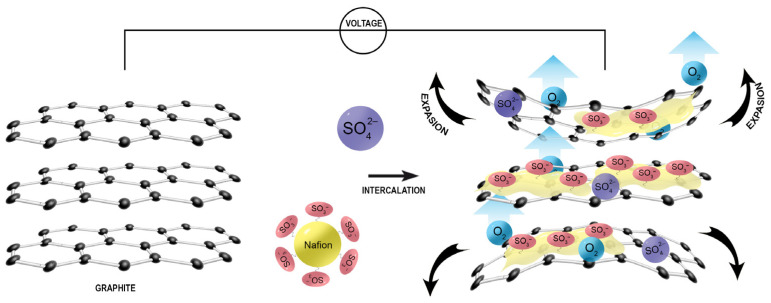
The representation of electrochemical exfoliation of graphite in the presence of Nafion.

Graphene and graphene materials are of interest due to their structure and characteristics. These are materials with a nearly two-dimensional structure, capable of existing in various forms that differ both in the number of layers and in the presence of various functional groups on the surface, which are easily controlled, for instance, via an electrochemical method of synthesis (Figure 3). Krasnova et al. [116] presented comparative results on the production of graphene with specified characteristics using various methods, including ultrasonic dispersion at different power levels and electrochemical exfoliation in different modes. The possibility of obtaining few-layer (1–3 layers) graphene with different oxygen content depending on the process conditions is reported.

The use of graphene and its modifications in combination with membranes has recently become widely spread both to increase humidity independence and to improve the overall set of properties [117].

Alnaqbi et al. [117] summarized recent progress in the application of graphene and its derivatives in various types of fuel cells, both as standalone materials and as modifiers of Nafion-based and other membranes. The review discusses their application in PEMFCs, DMFCs, anion-exchange DMFCs, and microbial fuel cells. In addition, engineering aspects of the application of these materials in different fuel cell systems are considered in detail. The importance of graphene and its derivatives for improving the performance of low-temperature fuel cells is highlighted.

The authors note the high efficiency of graphene in reducing fuel crossover. However, pristine GO-based membranes deteriorate proton-conducting properties as a result of the loss of oxygen-containing groups during proton transport. Sulfonation of GO partially solves this problem. Composite membranes based on graphene derivatives and Nafion-like membranes demonstrate the best combination of properties and stability.

Moreover, the following positive aspects of the use of graphene materials are reported: GO promotes water retention in the fuel cell membrane and shows high mechanical, chemical, and thermal stability. GO-based ionomer compositions also reduce fuel crossover. The application of graphene as a standalone membrane in PEMFCs was also summarized. The effectiveness of composite membranes containing iron (II,III) oxide and SGO as fillers was noted. Under dry conditions (25% RH and 120 °C), the composite membrane demonstrated a power density of 258.82 mW·cm^−2^, which is 80% higher than that obtained using a pristine recycled Nafion membrane.

Vinothkannan et al. [48] investigated a Nafion/Fe_3_O_4_–SGO composite membrane. The proton conductivity of the Nafion/Fe_3_O_4_–SGO (3 wt.%) composite membrane at 120 °C and RH of 20% was 11.62 mS·cm^−1^, which is 4.74 times higher than that of a pristine Nafion membrane. A PEMFC containing the Nafion/Fe_3_O_4_–SGO composite membrane delivered a peak power density of 258.82 mW·cm^−2^ at a current density of 640.73 mA·cm^−2^ when operating at 120 °C and 25% RH under atmospheric pressure. It should be noted that the use of compounds containing metal cations leads, in the long term, to degradation of the proton-conducting properties of the ionomer due to ion exchange of hydrogen ions with metal ions.

Mohamad Nor et al. [118] presented data on the effect of the amount of additive on the properties of a composite membrane. The addition of small amounts of sulfonated graphene enabled a fivefold increase in proton conductivity compared to a pristine Nafion membrane. However, the incorporation of more than 1.5% sulfonated graphene reduces proton conductivity values due to the excessive amount of graphene, which could disrupt the continuity of proton-conducting pathways. Thus, an optimal filler loading level is required to prevent deterioration of the mechanical integrity of the membranes and blockage of the proton conduction mechanism. Important factors include the size and distribution of the filler within the polymer matrix. Non-uniform distribution may lead to particle agglomeration and mechanical failure due to disruption of the homogeneity of the polymer film. The distribution of filler particles depends on chemical interactions between the polymer and the particles, particle size, and dispersion of the fillers in the casting solvent.

Basso Peressut et al. [119] proposed a simple method for preparing self-assembled SGO membranes (SGO-X), which are to be studied as a potential proton conductor for PEMFCs. The influence of three different molar ratios of sulfuric acid to GO is investigated, with the main aim of determining the optimal sulfonation range that ensures a successful compromise between composition, structural stability, and functional properties. ATR-FTIR and EDX spectroscopy, SEM, thermogravimetry, and static contact angle measurements were used to analyze the efficiency of GO functionalization with sulfonic acid groups (–SO_3_H) and the homogeneity of the component structure. A preliminary investigation of proton conductivity was carried out on the most promising samples (SGO-1, SGO-20) using electrochemical impedance spectroscopy, together with evaluation of water uptake, ion-exchange capacity, and degree of sulfonation. This work shows that the proposed SGO-X membranes exhibit noticeable water-retention and proton-exchange properties at elevated temperatures and reduced humidity compared to reference GO and Nafion 212 samples. Thus, these innovative standalone materials deserve further investigation to optimize their properties and to assess their behavior as a possible electrolyte in PEMFCs.

The use of graphene [30,43] and other nanostructured carbon materials [55] as stabilizers of Nafion has been investigated. It has been shown that in the Nafion/few-layer graphene composite, the strongest thermal stabilization of the ionomer occurs due to strong interfacial interaction between the components. Li et al. [120] presented the results of a study on the effect of incorporating GO polymer brushes as an inorganic additive together with Pt–TiO_2_ nanoparticles on humidity independence. This approach enabled the development of a new nanocomposite with a high level of self-humidification.

As noted above, the structure of the ionomer within the electrode plays a crucial role in determining its diffusion and electrochemical characteristics. In the electrode layer, the ionomer is typically present as thin films on the surfaces of carbon supports and metal nanoparticles. In some cases, controlled structuring of the ionomer into discrete agglomerates (island-like morphology) is applied [121]. This approach results in distinct effects on diffusion and proton-conducting properties: diffusion is enhanced due to the formation of transport pores, whereas proton conductivity may slightly decrease due to the formation of constricted pathways and reduced electrical contact between individual ionomer islands. It has been shown that, depending on the type of support, different structuring of the ionomer occurs [39,81,82]. The morphology of Nafion thin films on different carbon forms, as an analogue of fuel cell catalyst layers, has been studied. The issue of structure and proton transport in thin films (tens of nm) remains controversial due to the difficulty of experimental studies and interpretation of structural data. The studies conclude that different structures form on carbon and silicon surfaces with different hydrophilicity, leading to an inhomogeneous distribution of water across the film thickness.

The most commonly used materials for stabilizing proton conductivity include pristine graphene with surface functional groups, GO, and SGO.

The use of graphene and its derivatives for improving proton-exchange membrane properties follows general principles common to other material systems but also exhibits specific features related to the unique nature of graphene. A common approach involves introducing graphene-based materials as additives in amounts ranging from fractions to several percent in the membrane structure. Modification of properties occurs via two main mechanisms: water binding through hydrogen bonding of functional groups of graphene materials and improvement of the structure of proton-exchange channels in the membrane, including an increase in their diameter. Specific features of graphene use include the possibility of creating membranes from pristine GO or SGO, the possibility of flexible control over the structure and number of functional groups, and strong suppression of fuel crossover while maintaining proton conductivity.

A key advantage of graphene over metal-based additives lies in its inertness within the ionomer environment and the absence of ion-exchange processes that may lead to membrane degradation. The high surface area of graphene promotes strong interfacial interactions with Nafion molecules, resulting in a reduction in system energy and enhanced thermal stabilization of the ionomer. Graphene is highly effective in suppressing fuel crossover while preserving proton conductivity, which is essential for high-performance systems. However, particle segregation at elevated graphene loadings remains a challenge. Pristine GO-based membranes lose and deteriorate proton-conducting properties due to the loss of oxygen-containing groups during proton transport. Sulfonation of GO partially solves that problem. Composite membranes based on graphene derivatives and Nafion-like membranes demonstrate the best combination of properties and stability.

### 6.3. Critical Analysis of the Durability of Oriented, Nanocomposite and Surface-Engineered Membrane Architectures

The transition from isotropic distribution of additives to the creation of spatially oriented proton channels, supramolecular nanostructures, and membranes with modified surface topography represents a modern technological basis for overcoming the degradation limitations of PFSA (Nafion, Aquivion) and chitosan matrices. Quantitative life cycle analysis of such systems under accelerated climatic and mechanical tests reveals both significant advantages and hidden kinetic limitations.

1. Alignment kinetics and stability of oriented channels [108,111,114,115]

The creation of anisotropic, strictly directed proton-conducting pathways via magnetic field alignment, electrospinning, or block copolymer self-assembly fundamentally changes the nature of mechanical wear.

**Mechanical stability metrics:** Isotropic Nafion membranes under RH cycling exhibit volumetric swelling of 30–45% in all three axes. The introduction of magnetically oriented nanostructures or anisotropic electrospun fibers redistributes internal mechanical stresses. In-plane swelling of the membrane is reduced to a record low of 4–8%, which reduces viscoplastic fatigue by a factor of 3–5.

**Lifetime limit:** Such oriented systems successfully withstand up to 15,000–20,000 cycles according to US DOE standards without the formation of through-microcracks, retaining 92–95% of their original tensile strength. However, for block copolymers and nanostructures, the metastability of orientation remains a critical factor: at temperatures above 100 °C over long periods (>500 h), slow thermal relaxation of the chains is observed, leading to a decrease in conductivity anisotropy of 12–18%.

2. Durability of nanosheets, nanocomposites and carbon frameworks [107,112,113,116,117,119,122,123,124]

The integration of graphene nanosheets, sGO, and titanium dioxide-decorated multi-walled carbon nanotubes (TiO_2_-decorated MWCNTs) is aimed at simultaneous chemical protection and water retention under harsh conditions (up to 120 °C and 50% RH, per US DOE targets).

**Suppression of leaching and degradation:** Decorating carbon nanotubes with TiO_2_ nanoparticles or using Nafion as a surfactant during graphite exfoliation makes it possible to rigidly anchor the additive in the polymer matrix. The leaching rate of modified components decreases to trace values (<2–4% after 500 h of operation), whereas in classical binary mixtures this value reaches 30–40%.

**Anti-radical protection and FER:** Nafion/MWCNT and sGO composites effectively block the attack of •OH and •OOH radicals. During OCV hold tests at 90 °C, the FER decreases by a factor of 4–6 (to values ≤ 1 × 10^−6^ mg·cm^−2^·h^−1^) compared to pristine Nafion. This prevents radical “unzipping” of the chains and local membrane thinning. For chitosan membranes, sGO modification increases the thermal stability limit to 120 °C, preventing hydrolytic degradation of amino groups for 300+ hours of testing.

### 6.4. The Use of MOF

MOFs are used in proton-exchange membranes mainly as modifiers to improve their properties, such as proton conductivity, thermal and mechanical stability, as well as to reduce fuel permeability (crossover) [125,126,127]. Reviews have summarized the general principles of using MOFs as proton-exchange materials. MOFs, as porous inorganic–organic hybrid materials, have attracted considerable attention in gas storage, gas separation, and catalysis. Recently, MOF-modified DMFCs have demonstrated outstanding performance, highlighting their potential for commercial applications.

Annapragada et al. [128] classified and summarized the features of design, fabrication, performance characteristics, and practical applications of composite membranes using MOFs as fillers depending on the type of organic polymers. Future trends and development priorities were also considered and evaluated.

It has been noted that in the case of mixed matrix membranes based on MOFs, the interaction between the MOF and the polymer matrix not only creates new proton transport channels at the interface but also suppresses the movement of polymer chains, which enhances their thermal and mechanical stability compared to membranes made of pure polymer. At present, a number of MOF-based mixed matrix membranes with conductivity σ up to 10^−2^–10^−1^ S·cm^−1^ have been reported. Such composite membrane materials might have potential applications in the field of PEMFCs. In contrast, materials made of pure MOFs lack strength and toughness, hindering their independent application in fuel cells.

Liu et al. [126] presented a brief overview of proton conductivity in MOFs containing metal carboxylate, phosphonate, and sulfonate groups, as well as related structures, used as membranes in PEMFCs. Charge transport in these materials occurs through the Grotthuss mechanism. The hydrophilicity and acidity of the ligands are enhanced by functional groups –COOH, –PO_2_H, –SO_2_H, and –OH. Guest molecules and counterions facilitate the formation of hydrogen-bonded networks, which enhance proton conductivity. Various post-synthetic modifications and the described synergistic effects can be used to achieve exceptional conductivity, stability, and reliability. For the commercial use of MOFs in PEMFCs, several practical issues must be addressed, such as the operating temperature range, system reliability, interfacial flooding, manufacturability, electrode flooding by condensed water, crossover, and others. Although some of these challenges have been successfully addressed in MOF-based conductive materials, further research toward their commercialization is still ongoing.

#### 6.4.1. The Role and Advantages of MOFs in PEMs

- Enhancement of proton conductivity. MOFs may contain functional groups such as sulfonic (–SO_3_H) or carboxylic (–COOH) groups, which act as additional proton carriers or proton donors. Moreover, the porous structure of MOFs facilitates water retention, which is critically important for proton transport mechanisms (Grotthuss mechanism) and for maintaining high conductivity, especially at elevated temperatures or low humidity.
- Improved water retention. Embedding hygroscopic MOFs within a polymer matrix helps the membrane maintain a higher water content, which allows fuel cells to function across an extended temperature range without loss of efficiency.
- Barrier function. MOFs may alter the internal structure of a membrane by increasing its density and thereby hindering the diffusion of reactants (for example, hydrogen or methanol) through the membrane, which prevents crossover and enhances the overall efficiency of the fuel cell.
- Thermal and mechanical stability. Some MOFs exhibit high structural stability, enabling the fabrication of more durable membranes capable of withstanding fuel cell operating conditions.
- Morphology control. The use of MOFs as modifiers enables control over the morphology and free volume of composite membranes, thereby optimizing their transport properties.

MOFs are relatively new materials, and extensive research is currently focused on their potential applications in various areas, including proton-exchange materials. The promising nature of MOFs in this field is widely recognized.

The mechanism by which MOFs influence the stabilization of proton conductivity is similar to that of other membrane additives: improved water retention through water binding, enhancement of the ionomer structure, and its transport properties. By densifying the membrane structure, MOFs reduce reactant crossover.

At present, a number of mixed-matrix membranes based on MOFs with conductivities σ of up to 0.01–0.1 S·cm^−1^ have been reported. Such composite membrane materials are expected to have potential applications in the field of PEMFCs. As for membrane materials made of pure MOFs, which require supporting carriers, the lack of mechanical strength and viscosity makes their standalone use in fuel cells difficult.

#### 6.4.2. Critical Analysis of the Durability of MOFs and Ultrathin Ionomer Films

Investigation of thin-film analogues of catalytic layers and composite systems based on MOFs opens new pathways for radically reducing mass transport resistance and optimizing proton pathways. However, quantitative analysis of their long-term stability reveals specific degradation limitations associated with the confinement effect of ultrathin layers and the hydrolytic instability of framework coordination nodes [126,127,128].

1. Structural confinement and lability of ultrathin Nafion films on carbon [126]

The behavior of Nafion in the form of ultrathin films (thickness < 10–50 nm) deposited on carbon substrates differs radically from that of bulk membranes. These layers model the real three-phase boundary architecture in the fuel cell catalyst layer.

Confinement effect mechanism: Experimental data and X-ray and neutron reflectometry methods show that in ultrathin Nafion films, under the influence of the rigid carbon substrate, strong suppression of polymer chain mobility occurs. On one hand, this reduces free volume and decreases water content (the λ parameter drops from the usual 10–14 to a critical 3–4).

Quantitative degradation aspect: The restriction of macromolecular mobility leads to high lability of the ionomer structure under thermodynamic stress. Under the action of constant hydration-drying cycles and local heat generation, ultrathin Nafion films undergo accelerated mechanical relaxation. After 200–300 h of equivalent operation, irreversible ionomer shrinkage and a 15–25% change in its density are observed, which provokes an increase in oxygen mass transport resistance and local blocking of platinum catalyst active sites.

2. Problem of hydrolytic and chemical stability of MOF composites [126,127,128]

The integration of MOFs, such as the UiO, MIL, and ZIF families, into proton-exchange membrane matrices is one of the most promising directions for operation at high temperatures and low humidity due to their strictly ordered porosity and high concentration of proton donors within the pores.

Kinetic destruction of coordination bonds: The main factor limiting the durability of MOF membranes is their hydrolytic instability. The coordination bonds between metal ions (e.g., Zn^2+^, Cu^2+^) and organic linkers are vulnerable to attack by water molecules, especially under conditions of constant proton flow and elevated temperatures (>80 °C).

Quantitative durability metrics: Lifetime tests of Nafion/MOF composites without special chemical protection show that the coordination framework begins to degrade after only 100–150 h of operation. Destruction of the MOF structure leads to avalanche-like leaching of organic linkers and metal ions, as a result of which proton conductivity drops by 35–50%.

Catalyst poisoning: Leached free metal ions (Zn^2+^, Cu^2+^, Fe^3+^) migrate through the membrane to the catalytic layer. They act as Fenton reaction catalysts, accelerating the generation of destructive •OH and •OOH radicals by a factor of 3–5, which leads to a catastrophic increase in FER and through-hole perforation of the matrix.

3. Pathways to improve the durability of MOF systems

To exceed the stability threshold of 500–1000 h, current approaches focus on the use of chemically stable zirconium-based frameworks (UiO-66 family) or chromium-based frameworks (MIL-101), as well as on the method of covalent anchoring (chemical crosslinking) of MOFs to the polymer matrix.

Due to the strong Zr-O bonds in UiO frameworks and their functionalization with sulfonic groups (–SO_3_H), it is possible to reduce the leaching rate of components to trace values (<3–5% after prolonged AST tests). Such protected MOF composites retain up to 88–92% of their original power and structure under severe hygrothermal cycling conditions.

Thus, quantitative analysis of the literature data shows that when assessing the durability of ultrathin films and MOF composites, one cannot limit oneself to the standard wear mechanisms of bulk matrices. For ultrathin layers, the limiting factor becomes structural relaxation due to the confinement effect, while for MOF systems, the key issues are the hydrolytic stability of coordination nodes and the risks of catalytic poisoning of the MEA by their degradation products.

Table 4 compares advanced filler and morphology-control strategies that go beyond simple hydrophilic nanoparticle addition. These approaches are especially important because they attempt to control the geometry, continuity, and tortuosity of proton-conducting pathways rather than only increasing water uptake.

## 7. Quantitative Assessment of Strategies for Enhancing Humidity-Independence

Guided by the quantitative humidity-independence criteria introduced above (absolute conductivity ≥ 10 mS·cm^−1^ at 120 °C and 50% RH; conductivity decay ≤ 1–1.5 orders of magnitude upon RH reduction from 95% to 30%), a systematic analysis of the literature reveals that neat PFSA matrices do not meet the specified requirements. Upon dehydration, their conductivity drops exponentially by 2–3 orders of magnitude (falling well below 1 mS·cm^−1^) due to the loss of free-volume water and the structural collapse of isolated ionic channels.

In contrast, hybrid nanostructuring strategies employing 2D/3D functionalized fillers such as sSLM [3], MoO_2_ nanosheets [44], and organomodified graphene oxide [35] successfully achieve and surpass the US DOE benchmark. By modulating the local water activity coefficient and rigidly immobilizing bound water molecules at the interfacial boundaries, these hybrid architectures stably maintain absolute conductivity in the range of 12–45 mS·cm^−1^ under 30–50% RH at elevated temperatures.

Similarly, acid-doped alternative matrices (e.g., PBI) [27], by virtue of an anhydrous (hopping) conduction mechanism, completely decouple proton transport from dynamic environmental humidity fluctuations, formally satisfying the absolute DOE targets, although the justification for their application remains limited by the long-term leaching of liquid phosphoric acid.

### 7.1. Tuning Ionomer Structure via Various Pretreatment Methods

The adjustment of the structural organization of proton-conducting membranes at the pretreatment stage is a key tool for controlling their transport properties under dehydration conditions.

The architecture of the conducting channels is established during the preparation of ionomer dispersions in liquid media. Optimization of the solvent ratio allows control over the size of macromolecular aggregates and microphase separation during casting [69]. As shown in studies [61,62], precision assembly of the ionomer framework from optimized dispersions endows the membrane with an effective self-humidifying capability, enabling it to maintain an absolute conductivity of ≥15–20 mS·cm^−1^ when RH drops to 30%, which fully satisfies the low-temperature humidity-independence criterion.

However, thermal annealing of films at temperatures above the glass transition temperature (T_g_~110–130 °C) demonstrates an opposite, divergent trend with respect to the US DOE targets [70,71]. Activation of relaxation processes leads to recrystallization of the polymer backbone and densification of the hydrophobic domain packing [70,71]. On the one hand, this provides gains in stability and reduces methanol permeability by 50–70% [70]. On the other hand, shrinkage of the ionic domains causes an irreversible decrease in water content (λ parameter), reducing conductivity by 20–40% [25]. Atomistic molecular dynamics simulations confirm that annealing-induced chain densification acts as a steric barrier, reducing the rate of proton transport. Consequently, highly annealed neat PFSA membranes at 120 °C and 50% RH exhibit a conductivity drop below the 10 mS/cm threshold, failing to meet the high-temperature US DOE benchmark [25,72].

An even more critical performance decline is observed during hydrothermal aging under pressure in aqueous environments at temperatures above 100 °C, where coalescence of ionic clusters disrupts the percolation network and blocks the Grotthuss hopping mechanism [73]. A similar degradation trade-off between stability and conductivity is also characteristic of fluorine-free hydrocarbon alternatives, such as sulfonated SPEEK [73,75]. To prevent dissolution in water at elevated temperatures, SPEEK requires rigid thermal or chemical fixation of its structure. However, due to the high rigidity of the aromatic backbone and the inherently narrow channels (~1–2 nm), such pretreatment limits the conformational flexibility of the chains and reduces proton flux by 50–60%, shifting SPEEK conductivity deep into the sub-threshold region (<1–2 mS·cm^−1^) at 50% RH, rendering its commercialization impossible without the involvement of additional nanostructured hybrid additives.

### 7.2. Alternatives to Nafion Membranes

The development of fluorine-free and alternative aromatic polymer matrices represents a key strategy for overcoming the temperature barrier of Nafion and achieving the US DOE targets. Quantitative analysis of the literature shows that neat (unmodified) alternative hydrocarbon polymers are unable to overcome these barriers on their own, necessitating the use of specific doping and nanostructuring approaches.

For the class of sulfonated aromatic polymers (such as SPEEK and SPAES), the fundamental limitation is the strong dependence of transport on humidity, due to the channel tortuosity factor and weak hydrophobic–hydrophilic chain segregation [65,75]. To achieve the target conductivity at the DOE requirement level (10 mS·cm^−1^), neat hydrocarbon membranes require a high degree of sulfonation (DS > 60%), which at temperatures above 80 °C leads to catastrophic matrix swelling (>80–100%) and mechanical collapse [75]. The composite approach resolves this trade-off. Integration of 2D graphene oxide (GO) nanosheets into SPEEK and SPAES matrices allows fixation of the rigid polymer framework, reducing volumetric swelling from critical 60–80% to a stable 18–25% [30]. Due to the formation of an extensive hydrogen-bonding network at the polymer/GO interfaces, such composites smooth out the conductivity drop upon dehydration, successfully maintaining conductivity values at 15–35 mS·cm^−1^ under 30–50% RH, which fully satisfies the low-temperature humidity-independence benchmark [30]. The development of this concept includes electrospinning of SPEEK/GO fibers [100] and the creation of ternary multiblock fluorinated copolymers, where controlled nanostructuring ensures retention of conductivity above the 10 mS·cm^−1^ threshold even under substoichiometric conditions [31].

Alternative eco-friendly blend systems based on sulfonated poly(vinyl alcohol) (S-PVA) and poly(ether-block-amide) (PEBAX 1657), without modification, have unsatisfactory durability, losing mechanical stability in humid environments within just 10–24 h [78,98]. However, their targeted doping with titanium dioxide phosphate nanoparticles (TiO_2_PO_4_) [78] or crosslinking with inorganic sol–gel agents (TEOS) in SPEEK/PVA blends [99] dramatically changes the transport kinetics. Phosphated fillers act as local water-retention centers (stabilizing the λ parameter at ≥6 at low RH) [78], while the introduction of solid heteropolyacids (HPAs) generates additional stable conducting sites [95].

Ternary nanocomposite networks (including SPEEK/PVdF-HFP/SiO_2_ blends, where the fluoropolymer forms a chemically inert backbone) tightly confine bound water molecules at the interfacial boundaries [101], preventing dopant leaching and phase separation over 500–1000 h of testing. Such protected fluorine-free systems demonstrate stable conductivity in the range of 12–28 mS·cm^−1^ when humidity drops to 30%, confirming their applicability as humidity-independent membranes [101].

In the high-temperature range (120–200 °C), the technological basis for achieving US DOE targets is polybenzimidazole (PBI) doped with phosphoric acid (H_3_PO_4_) [14,96]. Due to the anhydrous hopping mechanism (prototropic transfer along the network of nitrogenous heterocycles), PBI/H_3_PO_4_ systems completely decouple proton transport from environmental humidity [77,96]. At high temperatures of 120–150 °C and under extremely dry conditions (50% RH or less), these membranes exhibit excellent absolute conductivity values in the range of 20–80 mS·cm^−1^, significantly exceeding the minimum DOE threshold of 10 mS·cm^−1^ [14,75].

Nevertheless, as emphasized in critical reviews of HT-PEMs, the justification for this strategy is limited by the progressive loss of mechanical viscosity upon excessive doping and the leaching of free acid by cathodic water vapor [14,75]. This causes a 40–60% drop in conductivity after the first 500 h of AST, shifting the system into the sub-threshold region [77]. This fact demonstrates that PBI membranes require the mandatory involvement of crosslinking agents or inorganic nanoframeworks (e.g., functionalized detonation nanodiamonds, analogous to advanced composites of the Aquivion type) for long-term fixation of proton-conducting pathways and prevention of capillary dopant leaching [32].

### 7.3. Oriented Structures and Composites, Including Those with Magnetic Properties

The creation of spatially oriented and anisotropic proton-conducting channels represents an advanced technological level of intensive nanostructuring. This strategy aims to minimize the tortuosity factor of transport pathways and drastically reduce mass-transfer resistance, which is critically important for maintaining membrane conductivity under severe dehydration conditions. Quantitative analysis of the literature shows that the transition from isotropic mixtures to molecularly oriented and nanostructured architectures allows not only achieving but also significantly exceeding these benchmarks [109,110,115].

Fundamental studies of ordered polymer nanosheets, multilayer structures, and supramolecular materials with controlled nanomorphology show that the strict organization of conducting pathways enables a reorganization of the proton transport mechanism [112,113,115]. In isotropic Nafion films, when humidity drops to 30%, channels narrow and become isolated, blocking transport. In contrast, molecularly oriented ultrathin polymer layers and biomimetic polyelectrolytes, due to the rigid fixation of functional groups, ensure continuous percolation even at extremely low λ levels [109,113]. Anisotropic alignment in defect-free membranes based on oriented block copolymers allows achieving in-plane conductivity on the order of 10^−2^–10^−1^ mS·cm^−1^ at equilibrium with moderately humid air, demonstrating complete independence of transport kinetics from macroscopic matrix swelling [111]. A similar breakthrough effect is demonstrated by electrospinning: anisotropically aligned electrospun nanofibers form a dense network of continuous “highways” for proton transfer, providing composite conductivity far beyond the minimum US DOE threshold of 10 mS·cm^−1^ under substoichiometric conditions [114].

The practical implementation of this concept into bulk membranes is realized through the integration of anisotropic carbon and inorganic frameworks, as well as methods for their alignment in external fields [107,108,115]. The incorporation of multi-walled carbon nanotubes decorated with titanium dioxide nanoparticles (TiO_2_-decorated MWCNTs) into a Nafion matrix creates a synergistic effect: hydrophilic TiO_2_ retains bound water at the molecular level, while MWCNTs act as a directing framework [107]. Such nanocomposites stably maintain conductivity at 15–40 mS·cm^−1^ under high-temperature and low-relative-humidity conditions, fully satisfying the high-temperature DOE benchmark.

The use of an external magnetic field for the forced alignment of stable proton-conducting channels (e.g., using magnetically oriented graphene oxide coated with Fe_3_O_4_, or other magnetic nanocomposites) allows fixing a strictly perpendicular or in-plane orientation of the transport pathways [108,115]. Magnetic ordering of the structure reduces the in-plane swelling ratio of the membrane to a record low of <6–8%, making it geometrically insensitive to environmental humidity fluctuations [115]. When RH drops from 95% to 30%, such magnetically aligned membranes maintain absolute conductivity values in the range of 25–65 mS·cm^−1^, exceeding isotropic Nafion by more than an order of magnitude [108,115].

Nevertheless, a critical quantitative analysis of long-term stability reveals hidden degradation risks of oriented magnetic systems. Under real fuel cell operating conditions, continuous liquid water generation at the cathode and local overheating stimulate slow electrochemical corrosion of the magnetic inorganic particles. The release of free metal cations (e.g., Fe^2+^/Fe^3+^) triggers irreversible ion exchange, replacing protons on the sulfonate groups, and catalyzes the Fenton reaction with an avalanche-like increase in fluoride emission rate (FER). This causes gradual macromolecular drift and thermal relaxation of the chains, as a result of which, after 300–500 h of AST tests, the anisotropic structure partially becomes disordered, and conductivity drops by 35–50%, shifting the system into the sub-threshold region.

Thus, the long-term stability of such systems is often limited by the gradual degradation of functional additives, driven by cation exchange or leaching of modifiers from the polymer matrix. A promising architectural strategy to address this issue involves a shift from simple composite blends toward the controlled design of inorganic–organic hybrid interfaces. A compelling example of this approach is the development of bilayer hybrid membranes of the type [Nafion/(WO_3_)_x_] [130]. The robust mechanical and chemical coupling between the organic and inorganic phases at the interface not only provides effective blocking of cation crossover (particularly vanadium ions in redox flow batteries) but also prevents the spatial degradation of the conducting domains. The formation of a stable, defect-free phase boundary rigidly fixes the spatial architecture of the hydrophilic pores, enabling uninterrupted proton transport over extended operational cycles. This points to a major pathway for overcoming the degradation of functional additives.

### 7.4. Application of Graphene-Based Materials

The integration of 2D carbon nanomaterials, such as graphene, GO, and sGO, represents one of the most intensively developing strategies for enhancing the humidity independence of PEMs [117]. Quantitative analysis of the literature shows that the unique geometry and surface chemistry of graphene derivatives enable these barriers to be successfully overcome, while simultaneously addressing the challenges of mechanical reinforcement and fuel crossover suppression [27,48].

Current progress in the field of graphene-based membranes for low-temperature fuel cells demonstrates that the key success factor is the restructuring of the ionic channel architecture [117]. The incorporation of sGO into flexible fluorine-free and hydrocarbon matrices (such as chitosan or sulfonated polyphenylsulfone sPPSF) dramatically alters transport kinetics. Due to the high density of intrinsic sulfonic acid groups (–SO_3_H) and a large specific surface area, sGO sheets form continuous anhydrous and low-hydration pathways for proton hopping via the Grotthuss mechanism. As documented in durability tests, self-assembling sulfonated graphene oxide membranes and chitosan/sGO-based composites maintain absolute conductivity at 15–45 mS·cm^−1^ in both fully hydrated and anhydrous states, significantly exceeding the minimum DOE threshold of 10 mS·cm^−1^ at low RH [118,119,124]. Similarly, the creation of crosslinked nanocomposite PEMs based on sulfonated polysulfone with graphene additives provides a self-humidifying effect, maintaining conductivity above the threshold of 12 mS·cm^−1^ at 30% RH [78].

Of particular scientific and practical interest are methods for the precision introduction of graphene materials into the bulk of Nafion ionomer. A comparative study of liquid-phase graphite exfoliation techniques using Nafion as a surfactant demonstrates that this approach allows obtaining highly stable suspensions without macro-aggregation of the nanosheets [116]. During composite casting, Nafion envelops the 2D carbon planes, fixing rigid interfacial boundaries. This strong interfacial entanglement effect reduces the conformational mobility of the ionomer chains, decreasing the in-plane swelling coefficient from an isotropic 45% to a stable < 10% [35,116]. This prevents viscoplastic deformation of the channels during dynamic humidity cycling (up to 20,000 cycles) [57].

This phenomenon is exacerbated in ultrathin Nafion films on carbon supports (thickness < 10–50 nm), which model the actual catalyst layer [82]. Under the influence of the confinement effect from the graphene or carbon framework, the mobility of the ultrathin ionomer chains is suppressed, causing the local λ parameter to drop to a critical value of 3–4, and the transport activation energy to increase to >0.4 eV. During long-term operation (exceeding 300 h), such ultrathin Nafion layers on carbon undergo local domain collapse and density compaction of 15–25% [125]. This leads to an irreversible drop in local conductivity below the threshold of 1–2 mS·cm^−1^, causing an avalanche-like increase in oxygen mass-transport resistance and limiting the MEA lifetime, necessitating mandatory covalent bonding or functionalization of the graphene interface to prevent degradation [61,62,125].

### 7.5. Application of MOFs

The integration of MOFs into polymer matrices for the construction of mixed matrix membranes (MMMs) represents a fundamentally new paradigm in the creation of humidity-independent electrolytes. A systematic analysis of the literature [126,127,128] shows that, thanks to their strictly regular three-dimensional porosity, high specific surface area, and flexible pore functionalization, MOF-based composites can exhibit unique transport properties that fully satisfy these numerical criteria.

Current progress in the development of proton-conducting membranes based on MOFs demonstrates their high effectiveness under severe dehydration conditions [126]. The incorporation of functionalized frameworks (such as the UiO-66, MIL-101, or ZIF families) with encapsulated sulfonic acid groups (–SO_3_H) or heteropolyacids into Nafion or alternative hydrocarbon polymer matrices allows achieving breakthrough performance. The regular pores of MOFs act as submicroscopic capillary reservoirs, which forcibly retain water molecules due to high capillary pressure, preserving the local hydration parameter λ ≥ 6 even when external RH drops to 30–50% [127]. As documented in experimental studies, mixed matrix membranes based on thermally stable zirconium frameworks (UiO-66-SO_3_H) provide absolute conductivity values in the range of 10^−2^ to 10^−1^ mS·cm^−1^ (12–85 mS·cm^−1^) at temperatures of 110–120 °C and 50% RH, significantly exceeding the minimum DOE threshold of 10 mS·cm^−1^ [126,127,128]. Moreover, the strictly ordered spatial distribution of MOF pores reduces the free volume of the polymer matrix and creates steric barriers to fuel crossover, reducing gas and methanol permeability by 45–60%, making these hybrids an ideal solution for high-performance systems [127,128].

However, when assessing the justification for employing MOF strategies, quantitative analysis of long-term stability reveals critical limitations. While nanostructured hybrid membranes successfully achieve high initial current characteristics, their durability during prolonged AST remains unsatisfactory. The pristine coordination nodes of most basic MOFs (metal-ligand bonds) exhibit pronounced fragility and are susceptible to hydrolytic degradation under the action of a continuous proton flux and elevated temperatures. After the first 100–150 h of operation under load, the coordination network begins to disintegrate, leading to avalanche-like leaching of organic linkers and a 35–50% drop in conductivity [126].

Furthermore, the degradation of the frameworks releases free metal ions, which migrate to the catalytic layers and provoke catalytic poisoning of the MEA [128]. This phenomenon is exacerbated when compared with the behavior of ultrathin Nafion ionomer layers on carbon supports (thickness < 10–50 nm), which model the catalyst layer [125]. Under the influence of the confinement effect from the rigid framework, the mobility of the ultrathin ionomer chains is suppressed, causing the local λ parameter to drop to a critical value of 3–4 and the transport activation energy to increase to >0.4 eV [125]. During long-term operation (exceeding 300 h), the ultrathin layers undergo local domain collapse and density compaction of 15–25%, driving conductivity deep into the sub-threshold region (<1–2 mS·cm^−1^). This demonstrates that to achieve commercial maturity (lifetime > 500–1000 h), MOF utilization strategies must be reoriented from simple physical doping to methods of covalent crosslinking or chemical anchoring of hydrolytically stable frameworks (UiO-66, MIL-101) within the polymer network.

## 8. Conclusions

This review has systematized the key scientific and technological strategies aimed at overcoming the critical barrier of modern PEMs, namely, their strong dependence on the degree of humidification (λ parameter) and rapid loss of stability under harsh operating conditions (up to 120 °C and 50% RH, per US DOE targets). A critical analysis of the data accumulated in recent years allows us to formulate the following key conclusions, identify fundamental knowledge gaps, and determine future research directions.

1. **Key materials science and “architectural” conclusions**
  − Three major directions for achieving substoichiometric humidification and humidity-independent conductivity have been systematized: the creation of organo-inorganic hybrid composites, solvothermal pre-equivalent tuning of the channel architecture of the polymer matrix, and the synthesis of alternative fluorine-free aromatic polymers (SPEEK, SPA, PBI).
  − It has been established that extensive enlargement of ionomer channel diameter through simple physical mixing of hydrophilic dopants is effective only in the initial stage (the first 100–200 h). In contrast, durable architectures resistant to cyclic degradation strictly require the creation of strong interfacial interactions.
  − Graphene derivatives, in particular sGO, have demonstrated the best balance between maintaining stable proton transport and suppressing fuel crossover. Due to their two-dimensional geometry and extensive interfacial surface with Nafion-like matrices, sGO minimizes free energy, increases thermal stability, and acts as a barrier framework, reducing anisotropic in-plane swelling of the membrane to values below 10%.
  − It has been demonstrated that the integration of MOFs within MMMs makes it possible to achieve high initial proton conductivity values (on the order of 10^−1^–10^−2^ S·cm^−1^) in intermediate- and high-temperature regimes. The stabilization mechanism is based on the strictly regular porosity of the frameworks, providing synergy between capillary water retention and orderliness of transport pathways.
  − The formation of spatially oriented, anisotropic proton channels (including under the action of magnetic fields, electrospinning methods, or block copolymer self-assembly) has been recognized as one of the most effective methods for reducing mass-transfer resistance and suppressing in-plane swelling.
2. **Identified knowledge gaps**
  − Deficit of predictive and systematic interfacial modeling: Despite an abundance of empirical data, the literature exhibits a severe deficit of comprehensive multiscale physical models that quantitatively link the thermodynamics of the ionomer/filler interface to the precise kinetics of hopping proton transfer under substoichiometric humidification conditions (λ < 3).
  − Chemical and structural degradation of carbon nanomaterials under proton flux: It has been established that during long-term proton transport, pristine graphene oxide (GO) undergoes gradual degradation and sacrificial reduction with loss of oxygen-containing functional groups. However, the precise kinetics of this process and its potential to catalyze radical “unzipping” of polymer chains remain unexplored.
  − Spatial segregation and phase drift of nanoparticles: The mechanisms governing local phase separation and macroaggregation of graphene fillers upon increasing their concentration in the PFSA matrix volume are still described qualitatively, without rigorous quantitative percolation thresholds for structural defects.
  − Hydrolytic instability of MOF nodes: The vast majority of pristine MOFs exhibit pronounced fragility, low fracture toughness, and complete hydrolytic instability of their coordination nodes (metal-linker bonds) under the action of a continuous proton flux. This renders their independent application without polymer supports impossible. The leaching kinetics of organic linkers during long-term operation (>100–150 h) remain a critical gap that is rarely assessed quantitatively.
  − Hidden risks of cation poisoning by magnetic additives: The use of magnetic inorganic modifiers carries a hidden degradation risk. In the long term (under conditions of liquid water generation and heating), electrochemical corrosion of magnetic particles occurs. The release of free metal cations triggers irreversible ion exchange (replacement of protons on sulfonate groups) and catalyzes the Fenton reaction, leading to avalanche-like destruction of the polymer matrix. The boundaries of chemical tolerance of ionomers to such cations are virtually unparameterized in the literature. A promising architectural strategy to address this problem involves a shift from simple composite blends toward the controlled design of inorganic–organic hybrid interfaces.
  − Structural relaxation of pretreated morphologies: Solvothermal pretreatment strategies are effective for initial channel tuning; however, due to the high conformational dynamics of perfluorinated chains, these artificial structures are susceptible to relaxation degradation. Under real operating conditions, thermal cycles and osmotic water transport force the labile channel system to relax back to the thermodynamically stable isotropic state, accompanied by domain shrinkage and a drop in conductivity.
3. **Trade-Offs of Alternative Matrices and the Supremacy of PFSA**
  − HT-PEM based on polybenzimidazole doped with phosphoric acid (PBI/H_3_PO_4_) satisfies most of the US DOE target indicators, with the exception of durability. The development of PBI matrices is strictly limited by the trade-off between maximizing initial conductivity, loss of mechanical strength upon excessive doping, and progressive leaching (“sweating”) of phosphoric acid by water vapor.
  − Quantitative comparison demonstrates that, due to their unique chemistry (fluorocarbon backbone, ultra-high acidity, low gas permeability, and ability to form dense monolithic films), Nafion-like ionomers remain the gold standard. Their destructive water leaching is minimal compared to the labile and hydrolytically unstable matrices of PVA, PVDF, or chitosan.
4. **Methodological gaps and future recommendations**
  − Deficit of fundamental mechanism analysis: The global literature is still dominated by isolated, empirical screening studies, where the mere fact of achieving short-term membrane functionality at low humidity is considered a positive result. Critically little attention is paid to the physicochemical mechanisms of λ parameter retention and the interfacial thermodynamics of additives, which limits the systematic design of materials.
  − Lack of standardized testing protocols: A severe methodological gap has been identified—the complete absence of a unified, generally accepted protocol for assessing the effectiveness of membrane humidity independence. Differences in temperatures, gas flow rates, and cell geometries among different research groups make a rigorous cross-comparison of strategies mathematically impossible. The absence of benchmarking standardization remains the main barrier to the transition of materials from laboratory scales to industrial commercialization.

In summary, it can be stated that the PEM industry is shifting from extensive modification methods to intensive nanostructuring. Three major directions—(1) three-dimensional magnetic or mechanical orientation of continuous proton pathways, (2) interfacial engineering using two-dimensional sGO, and (3) covalent anchoring of hydrolytically stable metal–organic frameworks (e.g., UiO-66)—represent the most viable technological basis. Future research should be focused not on maximizing initial conductivity, but on conducting standardized AST lasting at least 500–1000 h (including OCV hold and humidity cycling up to 20,000 cycles) to bridge the gap between laboratory metrics and real industrial durability.

Thus, overcoming the durability challenge of composite membranes requires a shift in research focus from mere screening of new materials toward addressing the fundamental issues of interface degradation. Based on the analysis conducted, the authors have formulated two key predictive directions for future research in this field:

1. Development and standardization of ASTs: For objective assessment of membrane durability, it is essential to implement unified protocols that combine cyclic humidity variation (from 100% to <20% RH) with controlled introduction of contaminating ions (e.g., Fe^2+^/Fe^3+^ or VO^2+^). This would enable in situ modeling of cation exchange processes and allow prediction of the actual service life of composites prior to commercial deployment.
2. Interface engineering to protect against cation poisoning: A promising strategy is the transition toward additives with firmly anchored functional groups and the creation of protective polymer nanolayers at the phase boundary. The formation of such barriers through covalent or coordination bonding would prevent the migration of metal cations to the sulfonate groups of Nafion, thereby eliminating blockage of proton channels and ensuring stable proton transport during prolonged operation.

## Figures and Tables

**Table 1:** Recent Nafion/PFSA-based composite membranes for conductivity stabilization under low humidity and elevated temperature.

Ref.	Membrane/Additive	Fabrication/Modification Route	Test Conditions	Main Conductivity/Performance Result	Proposed Stabilization Mechanism	Main Limitation
Loise & Simari, 2025 [3]	Nafion/sulfonated siliceous layered material, sSLM	Incorporation of sulfonated layered silica into Nafion matrix	120 °C, 20% RH; H_2_/O_2_ fuel cell	Power density increased to 340 mW cm^−2^ vs. 117 mW cm^−2^ for pristine Nafion	Hydrophilic sulfonated layers improve water uptake, ion-exchange capacity, dimensional stability and proton pathways	Long-term durability under cycling still needs deeper validation
Nicotera et al., 2025 [45]	Nafion/sulfonated clay–CNT, sCC	Hybrid sulfonated clay and CNT filler in Nafion	120 °C, 20% RH	Peak power density 443.2 mW cm^−2^, nearly fourfold higher than reprocessed Nafion	Clay improves water retention; CNTs improve mechanical integrity and conductive pathway formation	Possible aggregation and scalability of hybrid filler dispersion
Sun et al., 2025 [44]	Nafion/MoO_2_ nanosheets	Swelling–filling modification of Nafion with MoO_2_ nanosheets	PEM-relevant oxidative and conductivity tests	Oxidative stability improved by 56.7% and proton conductivity by 26% vs. Nafion	MoO_2_ acts as a radical scavenger and forms hydrogen bonds with Nafion chains	Metal-containing additives may require long-term ion-exchange/degradation assessment
Zhang et al., 2025 [47]	Nafion/NUS-9 sulfonated COF nanosheets	Incorporation of 0.5 wt.% sulfonated covalent organic framework nanosheets	PEMFC single-cell tests	Maximum power density 1.024 W cm^−2^, about 80% higherthan pristine Nafion	COF nanosheets introduce ordered sulfonated proton-conduction channels and water-retention sites	Durability and scale-up of COF synthesis remain key questions
Berber & Hafez, 2024 [33]	Nafion/phytic acid	Incorporation of phytic acid into Nafion	High temperature/low RH PEMFC-relevant conditions	Reported improvement in proton conductivity, chemical stability and fuel-cell performance	Phytic acid provides multiple phosphate groups for water binding and proton hopping	Acid leaching and long-term chemical stability should be verified under real PEMFC operation
Woo et al., 2024 [15]	Aquivion/sepiolite with fluorinated surface groups	Fluorine-containing grafting of sepiolite filler before incorporation into Aquivion-type membrane	RH below 30%	Membrane became less sensitive to RH decrease	Improved filler–ionomer compatibility and homogeneous filler distribution	Requires careful filler functionalization; limited long-term cell data
Primachenko et al., 2022 [32]	Aquivion/detonation nanodiamond, DND	0–5 wt.% positively charged DND in Aquivion-type PFSA membrane	22–120 °C, H_2_/O_2_ MEA tests	Improved current density and operational stability at elevated temperature	DND stabilizes PFSA morphology while preserving proton-conducting channels	Performance depends strongly on filler loading and particle distribution

**Table 2:** Comparative analysis of structural and proton-conductivity stabilization strategies for SPEEK-based membranes.

Modification Strategy	Key Achievement	Main Advantage	Limitation/Remaining Challenge
**2D fillers and carbon-based nanomaterials** Pg-C_3_N_4_, fluorinated graphite/graphene	Proton conductivity up to ~138 mS·cm^−1^; oxidative stability improved by up to 175%.	Enhanced oxidative protection, improved interfacial organization, and barrier effect against excessive swelling.	Possible aggregation of nanosheets at higher filler loadings, which may reduce membrane homogeneity and block proton-conducting pathways.
**Nanofibrous reinforcement** PI/SPEEK/PI frameworks	Conductivity loss of only 21% after six weeks, compared with 30% for Nafion 117 and 55% for pristine SPEEK.	Efficient suppression of dimensional deformation and improved long-term mechanical durability.	Increased membrane thickness and more complex fabrication of multilayer membrane architectures.
**Covalent cross-linking** Polyol-based cross-linking	Improved hydrolytic stability of the SPEEK matrix at elevated temperatures.	Restricts macromolecular chain mobility while preserving continuous proton-conducting pathways.	Possible decrease in proton conductivity due to partial consumption or reduced availability of free –SO_3_H groups.
**Ionic polymer blends** SPEEK/SPPS, SPEEK/SPPO	Ion selectivity approximately 2.5 times higher than that of Nafion 117.	Particularly promising for vanadium redox flow battery applications due to improved ion selectivity and reduced crossover.	Requires careful control of polymer compatibility and solvent selection to avoid phase separation.
**Morphology-induced self-organization** n-BuOH-assisted SPEEK	Peak proton conductivity of 314 mS·cm^−1^ at 80 °C.	Enables highly efficient proton transport through enlarged and better-connected ionic clusters.	Precise control of pore morphology, scalability, and membrane uniformity remains challenging.
**IL@MOF hybrid systems** UiO-66 with encapsulated ionic liquids	Stable proton conductivity in the range of 92–140 mS·cm^−1^.	Reduces ionic-liquid leaching and suppresses excessive water sorption, improving dimensional stability.	Multistep synthesis of host–guest hybrid fillers is relatively complex and labor-intensive.

**Table 3:** Nafion alternatives and non-fluorinated proton-conducting membranes.

Ref.	Membrane Class	Composition/System	Operating Window/Test Context	Key Result	Advantages vs. Nafion	Remaining Challenge
Qu et al., 2022 [77]	PBI-based membranes	PBI, acid-doped PBI, sulfonated aromatic polymers and composites	High-temperature PEMFCs	Review summarizes progress and challenges in HT-PEMs	Higher-temperature operation than hydrated Nafion; reduced external humidification requirement	Durability, acid retention and mechanical/conductivity balance remain unresolved
Das et al., 2024 [96]	Fluorine-free PEM	PBI, PBI mixed-matrix membranes, PBI-based ion-exchange membranes	Fuel cells, water electrolysis, desalination	Review covers structural modification, blending, cross-linking and organic–inorganic composites	High thermal stability and broad electrochemical applicability	Acid loss, long-term durability and mechanical degradation remain critical
Al-Mashhadani et al., 2025 [78]	Fluorine-free PEM	S-PVA/PEBAX 1657/TiO_2_PO_4_	80 °C, 80% RH PEMFC	Current density 175.5 mA·cm^−2^ and power density 61.52 mW·cm^−2^	PFSA-free composition; tunable water uptake and ion-exchange capacity through TiO_2_PO_4_ loading	Lower performance than optimized Nafion composites; long-term stability not fully established
Gomaa et al., 2024 [74]	Nafion/phytic acid	Cross-linked PVA/chitosan membranes sulfonated with dilute H_2_SO_4_	Reversible chlor-alkali electrochemical cell	PVA/CS 20 wt.% membrane outperformed Nafion in the tested system: 4 V vs. 6 V in electrolysis mode and 2.7 vs. 1.9 mW·cm^−2^ mgPt in fuel-cell mode	Non-fluorinated, low-cost and environmentally oriented membrane platform	Must still match PFSA durability, conductivity and chemical resistance
Zukova et al., 2025 [51]	SPEEK-based composite membrane	Non-fluorinated ion-exchange membranes for electrochemical applications	Water electrolysis and photoelectrochemical applications	Review frames material challenges for sustainable fluorine-free membranes	Addresses sustainability and PFAS-related concerns	Fluorine-free membranes still need to match PFSA membranes in conductivity, chemical stability, lifetime, and industrial scalability
Zhang et al., 2024 [102]	SPEEK-based composite membrane	Ultra-thin ordered SPEEK membrane fabricated by Langmuir–Blodgett self-assembly	Hydrated PEMFC-relevant proton-conductivity testing	The ordered SPEEK membrane showed proton conductivity of 0.384 S·cm^−1^, about three times higher than conventional SPEEK membranes	Low-cost hydrocarbon-based alternative; ordered molecular arrangement and ultrathin structure accelerate proton transport	Langmuir–Blodgett fabrication may be difficult to scale; long-term PEMFC durability, gas crossover, and mechanical robustness require further validation
Ben Ali et al., 2025 [103]	SPEEK-based composite membrane	SPEEK blended with partially oxidized polyvinyl alcohol, OPVA	Proton-conductivity testing up to 110 °C	SPEEK/OPVA with 20 wt.% OPVA reached 80 mS·cm^−1^ at 110 °C; OPVA also improved strength, stiffness, and thermal stability compared with pristine SPEEK	Non-fluorinated, low-cost membrane platform; hydrogen-bond network between OPVA and SPEEK provides additional proton-transfer sites	Increased water uptake may cause swelling; full-cell PEMFC performance and long-term hydrothermal/oxidative stability still need confirmation
Ponomar et al., 2024 [104]	PVDF-based sulfonated PEM	PVDF-SPA graft copolymer membrane	Compared with commercial PFSA and cation-exchange membranes	Systematic synthesis and physicochemical/ion-transport characterization of PVDF-based sulfonated PEM	Partially fluorinated alternative; cheaper and tunable through synthesis conditions	Requires further optimization of proton conductivity, swelling control, and chemical durability before practical PEMFC use
Mishra et al., 2024 [105]	PVDF-based modified PEM	O-PVDF-t-SSA centipede-type proton exchange membrane	PEM fuel cells/water electrolysis	Designed high-sulfonic-acid-density PVDF membrane for hydrogen-generation and fuel-cell applications	Combines PVDF stability with high density of proton-conducting groups	Stability of grafted/sulfonated structure under long operation must be confirmed
Komers et al., 2025 [50]	Sustainable PEMs for water electrolysis	Alternative PEM materials and challenges	Water electrolysis	Review discusses material requirements and sustainability challenges	Broadens the membrane discussion beyond fuel cells toward electrolyzers	Commercial adoption requires improved durability under electrolysis conditions, reduced gas crossover, and scalable low-cost fabrication
Kim et al., 2025 [62]	Self-humidifying PEMFC ionomer optimization	PFSA ionomers with optimized dispersion/catalyst-layer structure	Portable air-breathing PEMFCs	Ionomer optimization improves self-humidifying PEMFC performance	Shows that ionomer morphology control can reduce external humidification demand	More relevant to catalyst layer than standalone membrane; durability data needed
Safronova et al., 2022 [69]	PFSA morphology control	Nafion cast from different dispersion liquids	Membrane casting from water–alcohol and aprotic solvents	Solvent-controlled morphology affects water uptake, mechanical properties and conductivity	No new filler required; scalable processing route	Pretreatment-induced morphology may relax during long operation

**Table 4:** Advanced nanostructured fillers and morphology-control strategies reported in the recent literature.

Ref.	Membrane Class	Composition/System	Key Structural Idea	Reported Result	Mechanistic Interpretation
Shao et al., 2023 [129]	Sulfonated COF nanochannels	ZUT-COF-SO_3_H@Nafion	2D COF with multichannel proton-conduction structure	Proton conductivity 0.1338 S·cm^−1^, activation energy 0.086 eV, power density 304.056 mW cm^−2^	COF nanochannels and high sulfonate loading provide additional proton-transport pathways
Zhang et al., 2025 [47]	Sulfonated COF nanosheets	Nafion/NUS-9	Ultrathin sulfonated nanosheets inside Nafion	Maximum power density 1.024 W·cm^−2^, about 80% higher than pristine Nafion	Reduced proton-transport barriers and increased proton donor density
Nicotera et al., 2024 [107]	TiO_2_-decorated MWCNTs	Nafion/MWCNT–TiO_2_	Long CNTs provide extended pathways; TiO_2_ retains water	NMT-3 reached 307.7 mW·cm^−2^ at 120 °C and 30% RH	CNTs assist path continuity; TiO_2_ supports hydration under dehydrating conditions
Alnaqbi et al., 2024 [117]	Graphene-based membranes and fillers	Graphene, GO, SGO in Nafion and non-Nafion PEMs	2D barrier/conductive filler with tunable oxygen/sulfonic groups	Review reports improved fuel-cell performance and reduced fuel crossover	Graphene derivatives retain water, suppress crossover, and improve mechanical/thermal stability
Vinothkannan et al., 2018 [48]	Magnetic SGO hybrid filler	Nafion/Fe_3_O_4_–SGO	Magnetic oxide + sulfonated GO hybrid	Conductivity 11.62 mS·cm^−1^ at 120 °C, 20% RH, 4.74× higher than recast Nafion	SGO improves water uptake and proton transfer; Fe_3_O_4_ modifies ionic domains
Randall et al., 2024 [125]	Thin-film ionomer morphology	Nafion thin films on carbon supports	Catalyst-layer analogue; morphology depends on carbon surface	Demonstrated substrate-dependent Nafion morphology in thin films	Interfacial interactions affect hydration and proton transport in catalyst layers
Liu et al., 2022 [126]	MOF-based PEMs	MOF/polymer mixed-matrix membranes	MOF pores and acidic ligands form proton-conducting networks	Review reports MOF-based mixed-matrix membranes with conductivity up to 0.01–0.1 S·cm^−1^	Hydrophilic pores, acidic ligands, guest molecules and counterions support Grotthuss-type transport
Annapragada et al., 2023 [128]	MOF mixed-matrix membranes	MOF/polymer PEMs	MOF fillers classified by matrix type and membrane design	MOFs identified as promising fillers for PEMFC membranes	MOF–polymer interfaces create additional proton-transport channels and improve stability

## Data Availability

No new data were created or analyzed in this study. Data sharing is not applicable to this article.

## Abbreviations

The following abbreviations are used in this manuscript:

AST	Accelerated stress testing
CNT	Carbon nanotube
CS	Chitosan
DMFC	Direct methanol fuel cell
DND	Detonation nanodiamond
DOE	Department of Energy
EW	Equivalent weight
FER	Fluoride emission rate
GO	Graphene oxide
HFP	Hexafluoropropylene
HPA	Heteropolyacid
HT-PEM	High-temperature proton exchange membrane
HT-PEMFC	High-temperature proton-exchange membrane fuel cell
MEA	Membrane–electrode assembly
MD	Molecular dynamic
MOF	Metal–organic framework
NMR	Nuclear magnetic resonance
OCV	Open-circuit voltage
PBI	Polybenzimidazole
PEMFC	Proton-exchange membrane fuel cell
PFSA	Perfluorosulfonic acid
PVA	Polyvinyl alcohol
PVDF	Polyvinylidene fluoride
RH	Relative humidity
SAXS	Small-angle X-ray scattering
sCC	Sulfonated Clay-Carbon nanotubes
SEM	Scanning electron microscopy
SFMC	Sulfonated fluorinated multiblock copolymer
SGO	Sulfonated graphene oxide
SPA	Sulfonated poly(arylene ether)
SPAES	Sulfonated poly(arylene ether sulfone)
SPEEK	Sulfonated poly(ether ether ketone)
SPPS	Sulfonated polyphenylene sulfide
sSLM	Sulfonated layered silica material
TEOS	Tetraethoxysilane
QENS	Quasi-elastic neutron scattering
